# Harnessing the Anti-Nociceptive Potential of NK2 and NK3 Ligands in the Design of New Multifunctional μ/δ-Opioid Agonist–Neurokinin Antagonist Peptidomimetics

**DOI:** 10.3390/molecules26175406

**Published:** 2021-09-06

**Authors:** Charlène Gadais, Justyna Piekielna-Ciesielska, Jolien De Neve, Charlotte Martin, Anna Janecka, Steven Ballet

**Affiliations:** 1 Research Group of Organic Chemistry, Departments of Bioengineering Sciences and Chemistry, Vrije Universiteit Brussel, Pleinlaan 2, 1050 Brussels, Belgium; jolien.de.neve@vub.be (J.D.N.); charlotte.martin@vub.be (C.M.); 2 Institut des Sciences Chimiques de Rennes, Equipe CORINT, UMR 6226, Université de Rennes 1, 2 Avenue du Pr. Léon Bernard, CEDEX, 35043 Rennes, France; 3 Department of Biomolecular Chemistry, Faculty of Medicine, Medical University of Lodz, 92-215 Lodz, Poland; justyna.piekielna@umed.lodz.pl (J.P.-C.); anna.janecka@umed.lodz.pl (A.J.)

**Keywords:** peptidomimetics, designed multiple ligand, peptide, μ-opioid receptor, δ-opioid receptor, opioid agonist, neurokinin, NK2, NK3, antinociception

## Abstract

Opioid agonists are well-established analgesics, widely prescribed for acute but also chronic pain. However, their efficiency comes with the price of drastically impacting side effects that are inherently linked to their prolonged use. To answer these liabilities, designed multiple ligands (DMLs) offer a promising strategy by co-targeting opioid and non-opioid signaling pathways involved in nociception. Despite being intimately linked to the Substance P (SP)/neurokinin 1 (NK1) system, which is broadly examined for pain treatment, the neurokinin receptors NK2 and NK3 have so far been neglected in such DMLs. Herein, a series of newly designed opioid agonist-NK2 or -NK3 antagonists is reported. A selection of reported peptidic, pseudo-peptidic, and non-peptide neurokinin NK2 and NK3 ligands were covalently linked to the peptidic μ-opioid selective pharmacophore Dmt-DALDA (H-Dmt-d-Arg-Phe-Lys-NH_2_) and the dual μ/δ opioid agonist H-Dmt-d-Arg-Aba-βAla-NH_2_ (KGOP01). Opioid binding assays unequivocally demonstrated that only hybrids **SBL-OPNK-5**, **SBL-OPNK-7** and **SBL-OPNK-9**, bearing the KGOP01 scaffold, conserved nanomolar range μ-opioid receptor (MOR) affinity, and slightly reduced affinity for the δ-opioid receptor (DOR). Moreover, NK binding experiments proved that compounds **SBL-OPNK-5**, **SBL-OPNK-7**, and **SBL-OPNK-9** exhibited (sub)nanomolar binding affinity for NK2 and NK3, opening promising opportunities for the design of next-generation opioid hybrids.

## 1. Introduction

From the ancient use of the plant alkaloid morphine up until the discovery of modern medicine technologies, opioid receptor-targeting analgesics still occupy a prominent place in the management of acute or chronic pain. Through a competition with endogenous neuropeptides (e.g., endorphin, enkephalins, and dynorphins), these small molecule drugs work by primarily activating the μ-opioid receptor (MOR) [1], and to a lesser extent, the δ-opioid (DOR), κ-opioid (KOR), and nociceptin (NOP) receptors. Aside from their pivotal role in nociception, these receptors modulate other vital biological processes such as respiration, gastrointestinal transit, stress responses, neuroendocrine, and immune functions [2,3]. This variety in physiological processes, combined with the multitude of neurotransmitters and associated receptors involved in pain pathways, explain the challenges that have to be overcome when designing new analgesic drugs. Decades of research unveiled that despite their undeniable efficiency in pain control, prolonged use of opioid drugs can cause severe adverse effects being detrimental to the life quality of treated patients [4,5]. More precisely, it is notorious that opioid analgesics participate in cancer progression, but also cause respiratory depression and hyperalgesia [6]. Additionally, visceral disorders such as constipation, nausea, and emesis can be cited in the list of the physically disabling symptoms [7,8], while accumulating evidence link mood variation [9], reward processing, and psychosocial disorders to the consumption of opioid drugs [10]. To tackle these side effects and to improve patient life quality, polypharmacological approaches including drug cocktail administration have been scoped in past years. Such clinical approaches, nonetheless, have raised new challenges such as the control of drug–drug interactions, complex pharmacokinetic profiles, or poor patient compliance. Access to alternative therapeutic strategies is therefore highly demanded. 

Defined as a single chemical entity bearing two pharmacophores with affinity and selectivity toward two (or more) distinct molecular targets, designed multiple ligands (DMLs) aim at improved pharmacokinetic/pharmacodynamic and safety profiles compared to combination therapies. Treatment with a unique molecule drastically simplifies therapeutic procedures and allows for the overall doses to be lowered. Such multitarget-directed ligands are thus anticipated to balance therapeutic efficacy and bioavailability, while minimizing minor to severe and harmful side effects [11]. More specifically, when rationally designed to target pain management by activating the μ-opioid (and possibly δ-opioid receptors) through one of the DML’s pharmacophores, the second motif is commonly aimed to compensate for side effects by modulating other non-opioid receptors involved in pain signaling pathways [12]. More precisely, the second pharmacophore is usually intended to lessen respiratory depression, emesis, drug-related tolerance, physical and psychological dependence, all being inherently associated with opiate drug administration. 

Via the pioneering discovery of Substance P (SP), a whole family of endogenous neuropeptides was unveiled and rapidly considered as an attractive target for brain-related disorders. The neurokinin ligands NKA and NKB, in particular, and their preferred transmembrane receptors, neurokinin-2 (NK2) and neurokinin-3 (NK3), respectively, both belonging to the GPCR family, are essentially present in the central nervous system (CNS) and the periphery, where they exert neuromodulatory activities in a wide range of patho- and physiological processes [12,13]. Their involvement in visceral nociception and inflammation, pain memory, and pain-induced stress response as well as neuroendocrine disorders motivated the design of several peptidic and non-peptidic drugs targeting NK2–NK3 receptors [13,14,15].

**Figure 1:**
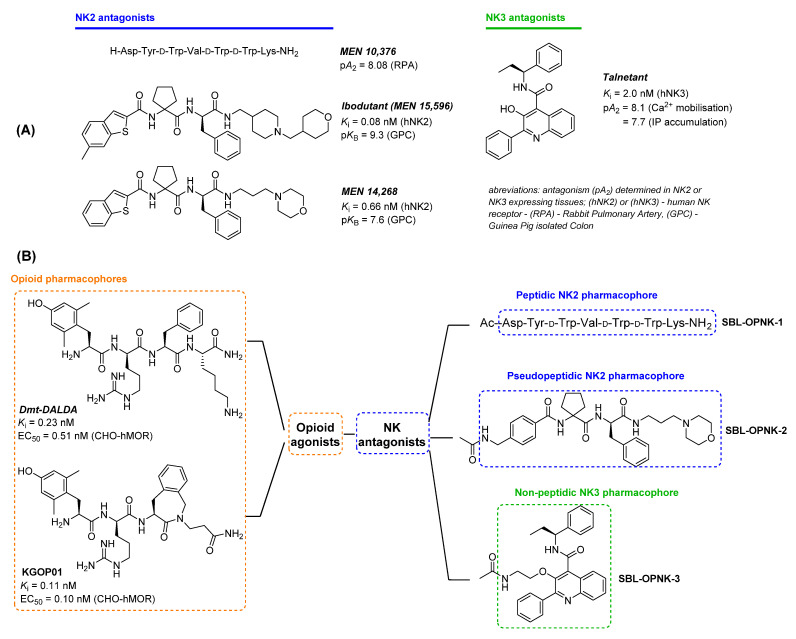
(**A**) Selection of high affinity neurokinin NK2 and NK3 antagonists with peptidic, pseudopeptidic, and non-peptidic scaffolds. (**B**) Design of opioid agonist–NK2/NK3 antagonist hybrids and molecular structure of composing pharmacophores.

Considering both the non-exclusive interaction of the three neurokinin ligands with their receptors and their common involvement in major physiological events, the close synergistic activity between the opioid and neurokinin systems was recently reviewed [16]. Notably, a major interest in SP as a potent target in pain treatment was largely reflected by the numerous studies published in the last decade(s) [13,17,18]. Recently, our group also developed opioid agonist–neurokinin NK1 antagonist hybrids, which displayed promising activity in neuropathic pain models [19,20]. However, unlike NK1, hybrid opioid-NK2 and -NK3 drugs aiming at nociception and opioid-induced adverse effects regulation have been barely investigated, despite multiple reports of opioid-NK2/NK3 crosstalk. Indeed, the involvement of the NKB/NK3 system in various physiological processes including pain hypersensitivity and visceral nociception was recently highlighted [21]. Within the same context, the role of the NKA/NK2 system was also compared to the opioid and SP/NK1 system [22]. However, the exact mechanism of interaction of all those actors in nociception remains to be elucidated. Hence, the design of molecules targeting both the opioid receptors and either the NK2 or NK3 receptor would provide additional insights to pain or pain-related treatments, while helping in deciphering the role of NK2 and NK3 receptors in the regulation of the opioid system. In this work, a first series of multifunctional opioid agonist–neurokinin NK2 or NK3 antagonist ligands were designed, synthesized, and evaluated in vitro in light of exploring new therapeutic pathways in pain and related disorders. Reported for their high MOR binding affinity [23,24], the two putative opioid agonist pharmacophores Dmt-DALDA and KGOP01 were covalently linked to a selection of peptidic, pseudo-peptidic, and non-peptidic NK2 and NK3 pharmacophores **SBL-OPNK-1**, **SBL-OPNK-2**, and **SBL-OPNK-3** (Figure 1B). As described below, the design of these neurokinin-targeting moieties was inspired by the structures of reported and approved NK2 and NK3 antagonists exhibiting good biological activity. The first-generation peptidic NK2 ligand MEN 10,376 (Figure 1A) displayed good antagonistic activity at the NK2 receptor (p*A*_2_ = 8.08 ± 0.1 in RPA–endothelium-deprived rabbit pulmonary artery against neurokinin A as an agonist), hence offering an adequate peptide-based candidate for this study [25]. Exhibiting subnanomolar and selective NK2 binding affinity, and investigated in clinical phases for irritable bowel syndrome treatment [26], Ibodutant, and one of its parents, MEN 14,268, were selected as starting scaffolds for peptidomimetic NK2 ligand design (Figure 1A). Finally, as a major representative of the NK3 antagonist family, and as a compound examined in clinical trials for treatment of CNS disorders, Talnetant exhibited high affinity for human NK3 and long-lasting in vivo activity, justifying its selection as a starting scaffold for non-peptidic NK3 ligand design [27]. As the first challenge in multifunctional analgesic ligand design is to maintain affinity to both targets and agonist efficacy at the opioid receptors, this initial report aimed to investigate the binding affinity and opioid receptor activation of the newly designed opioid-NK2 and -NK3 hybrids. 

## 2. Results

### 2.1. Chemistry

**Figure 2:**
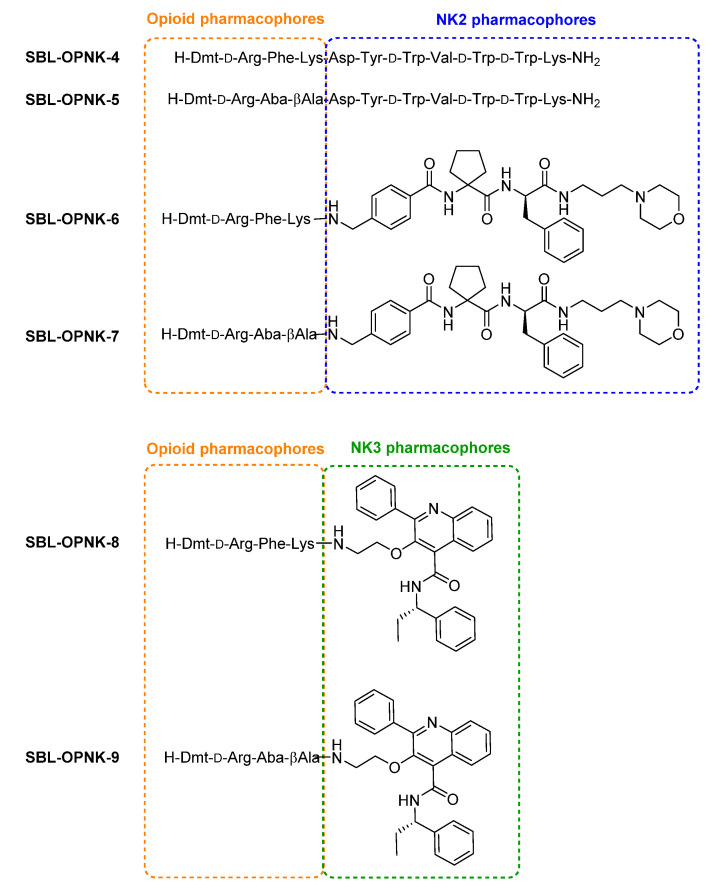
Designed opioid agonist-neurokinin NK2 antagonist and opioid agonist-neurokinin NK3 antagonist hybrids.

The structures of the targeted opioid agonist–neurokinin NK2 or NK3 antagonist ligands are presented in Figure 2. 

**Scheme 1:**
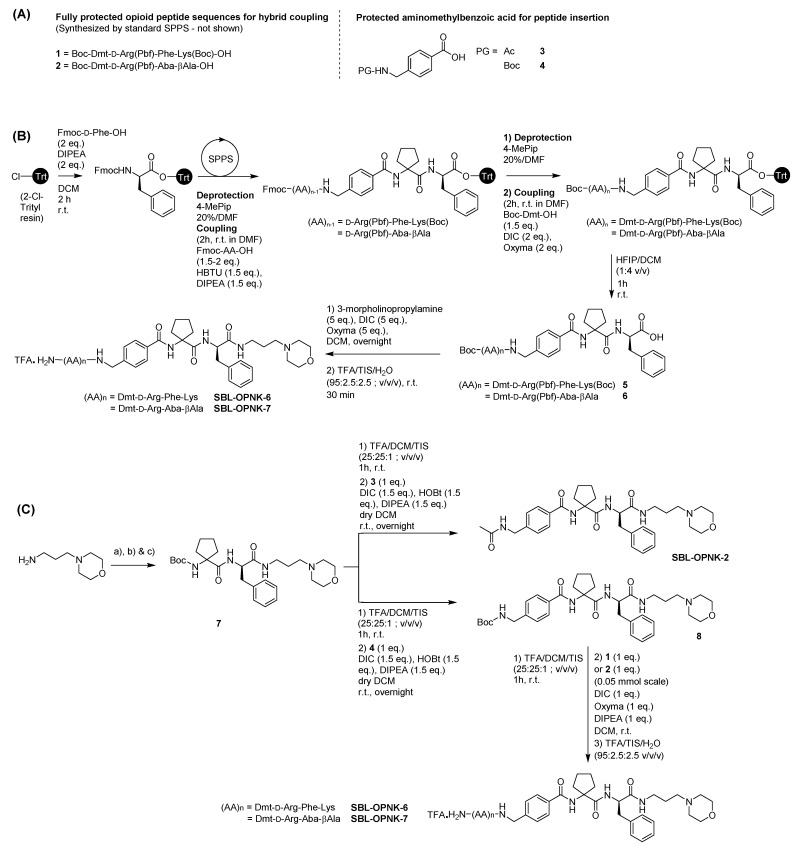
(**A**) Sequences of fully protected opioid units **1** and **2** for hybrid coupling (left) and protected aminomethylbenzoic acid structures **3** and **4** for peptide insertion (right). (**B**) Solid-phase assembly of the opioid and neurokinin pharmacophores, and solution phase coupling of the resulting cleaved hybrid intermediates to the *C*-terminal morpholine moiety. Reaction conditions on 0.15 mmol scale and 2-chlorotrityl chloride resin (loading 0.76 mmol/g). (**C**) Solution phase assembly was initiated at the 0.2 mmol scale and the last coupling was carried out at the 0.05 mmol scale. (a) Boc-d-Phe-OH (1 eq.), DIC (1.5 eq.), HOBt (1.5 eq.), DIPEA (1.5 eq.), dry DCM r.t., overnight; (b) TFA/DCM/TIS (25:25:1; *v*/*v*/*v*) 1 h, r.t.; (c) Boc-cycloleucine (1 eq.), DIC (1.5 eq.), HOBt (1.5 eq.), DIPEA (1.5 eq.), dry DCM r.t., overnight.

Peptide-based pharmacophores Dmt-DALDA, KGOP01, and **SBL-OPNK-1**, and peptide-based hybrids **SBL-OPNK-4** and **SBL-OPNK-5** were synthesized following standard solid phase peptide synthesis (SPPS) procedures on rink amide resin to obtain *C*-terminal carboxamides (not shown). Hybrid compounds **SBL-OPNK-6** and **SBL-OPNK-7** were synthesized either by first building the linear peptide scaffold on 2-chloro-trityl chloride resin, following standard SPPS, cleavage from the resin, and final coupling of 3-morpholinopropylamine in solution (Scheme 1B), or by sequential solution-phase couplings starting from 3-morpholinopropylamine (Scheme 1C). In this strategy, fully protected Boc-Dmt-d-Arg(Pbf)-Phe-Lys(Boc)-OH **1**, and Boc-Dmt-d-Arg(Pbf)-Aba-βAla-OH **2** (Scheme 1A) were first synthesized by SPPS, as reported elsewhere [28]. For both strategies and when necessary, commercially available 4-(aminomethyl)benzoic acid, and cycloleucine were adequately *N*-protected following standard procedures, before their insertion (Scheme 1A and Supplementary Materials). 

**Scheme 2:**
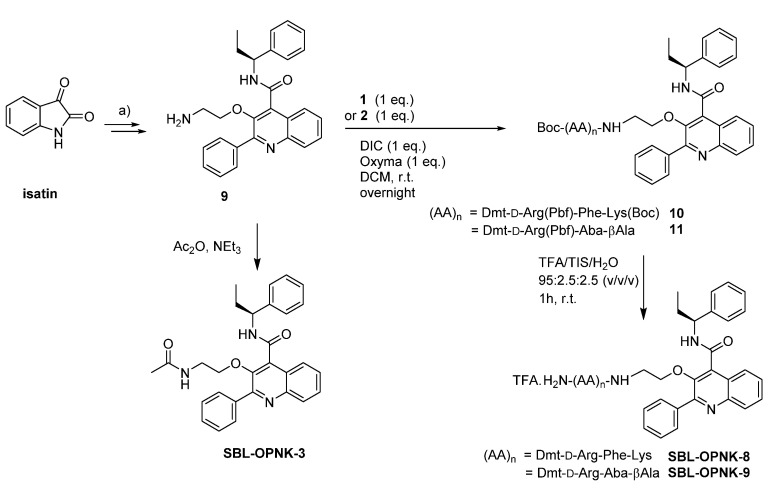
Synthetic path toward the NK3 ligands. For (a) all steps are detailed in the Supplementary Materials.

The non-peptidic NK3 pharmacophores were obtained by first preparing intermediate **9** following the reported procedures starting from isatin (Scheme 2 and Supplementary Materials) [29]. In a next step, **9** underwent acetylation to give **SBL-OPNK-3** or peptide coupling with **1** or **2** to give the desired hybrid compounds **SBL-OPNK-8** and **SBL-OPNK-9** (Scheme 2).

### 2.2. Biological Evaluation

#### 2.2.1. Opioid Receptor Affinity Assay

Compounds Dmt-DALDA and KGOP01, known as potent opioid receptor ligands [19,23], were used as reference pharmacophores. Dmt-DALDA was reported to possess strong, subnanomolar, affinity, and selectivity for MOR, while compound KGOP01 binds to MOR in subnanomolar and to DOR in the nanomolar range. Both reference compounds showed only weak binding at KOR. Hybrid compounds **SBL-OPNK-4**, **SBL-OPNK-5**, **SBL-OPNK-7**, and **SBL-OPNK-9** displayed slightly reduced MOR affinity in a low nanomolar range. Weak binding to MOR was observed for **SBL-OPNK-6** and **SBL-OPNK-8**. Compounds **SBL-OPNK-7** and **SBL-OPNK-9** were strong DOR ligands (*K*_i_ = 3.26 and 6.57, respectively), compound **SBL-OPNK-5** showed an order of magnitude decreased affinity, whereas **SBL-OPNK-4**, **SBL-OPNK-6**, and **SBL-OPNK-8** did not bind efficiently to DOR (Table 1). All tested compounds showed very weak binding to KOR. 

#### 2.2.2. Calcium Mobilization Assay

**Figure 3:**
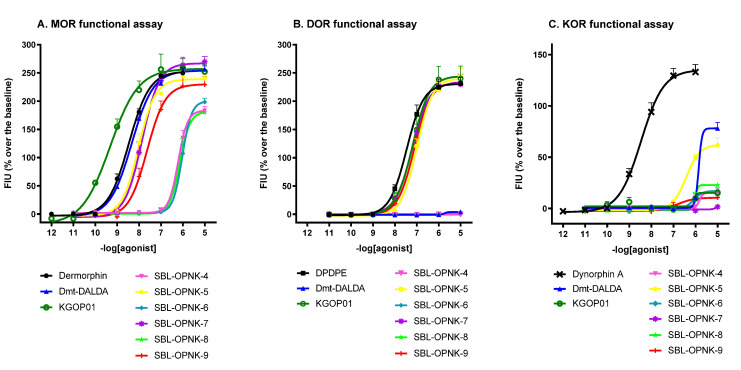
Concentration–response curves for standard and tested compounds in calcium mobilization experiments performed in: CHO MOR cells stably expressing the Gα_qi5_ protein (Panel **A**), CHO DOR cells stably expressing the Gα_qG66Di5_ protein (Panel **B**), and CHO KOR cells stably expressing the Gα_qi5_ protein (Panel **C**). Data are shown as the mean ± SEM of at least five separate experiments performed in duplicate.

**Figure 4:**
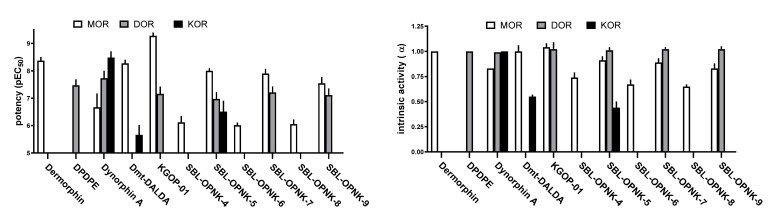
Potencies (left panel) and efficacies (right panel) of the tested compounds at opioid receptors. Potency values are expressed as mean ± CL_95%_ while intrinsic activity values are expressed as mean ± SEM.

In the calcium mobilization assay, all compounds were assessed under the same experimental conditions and their effects were compared to the reference compounds: dermorphin, DPDPE, and dynorphin A for MOR, DOR, and KOR, respectively (Table 2 and Figure 3 and Figure 4).

At MOR, compound Dmt-DALDA mimicked the stimulatory effect of dermorphin in the calcium release test and KGOP01 showed full efficacy, but potency about 8-folds higher than the standard compound (α = 1.04, EC_50_ = 0.54). Hybrids **SBL-OPNK-5** and **SBL-OPNK-7** showed slightly reduced potencies compared to the reference compound dermorphin (2- and 3-fold, respectively) and to the opioid parent pharmacophore KGOP01 (18- and 24-fold, respectively). Hybrid **SBL-OPNK-9** moderately stimulated calcium release, while the remaining compounds showed strong (>200-fold) reduction in potencies compared to dermorphin (Table 2 and Figure 3A). With regard to DOR agonism, compounds KGOP01, **SBL-OPNK-5**, **SBL-OPNK-7**, and **SBL-OPNK-9** evoked calcium signaling responses with a stimulatory effect of the reference DPDPE, but with 2–3-fold decreased potencies (Table 2 and Figure 3B). As for potency at KOR, compounds Dmt-DALDA and **SBL-OPNK-5** were able to elicit a weak stimulatory response at micromolar concentrations. Compound **SBL-OPNK-7** was completely inactive, while the other tested compounds weakly stimulated calcium release only at the highest dose (10 μM), providing incomplete concentration–response curves (Table 2 and Figure 3C).

#### 2.2.3. Neurokinin Receptors Binding Affinity Assay

In light of the opioid binding and functional assay data, hybrids **SBL-OPNK-5**, **SBL-OPNK-7**, and **SBL-OPNK-9** were selected for further in vitro biological evaluation and compared to the corresponding neurokinin parent pharmacophores **SBL-OPNK-1**, **SBL-OPNK-2**, and **SBL-OPNK-3**, respectively. Neurokinin receptor binding assays were carried out using [Nle^10^]-NKA(4-10) and SB 222200 as NK2 and NK3 reference ligands. In the NK2 radioligand binding assay, *IC*_50_ and *K*_i_ values could only be calculated for the peptidic NK2 pharmacophore **SBL-OPNK-1**, with about 17-fold lower binding affinity compared to the reference compound. The selected hybrids, however, showed promising results. Compounds **SBL-OPNK-5** and **SBL-OPNK-7** possessed improved binding at the NK2 receptor compared to the NK2 parent pharmacophores **SBL-OPNK-1** and **SBL-OPNK-2**. While compound **SBL-OPNK-5** displayed nanomolar affinity, **SBL-OPNK-7** presented an even stronger, sub-nanomolar affinity (Table 3). 

In the NK3 radioligand binding assay, the parent NK3 pharmacophore **SBL-OPNK-3** showed a slightly reduced affinity compared to the reference compound, while the corresponding hybrid ligand **SBL-OPNK-9** presented again a 1.5-fold increase of its NK3 affinity and potency compared to the reference compound SB 222200.

## 3. Discussion

To address the side effects associated with the long-term use of opioid agonists, the design of multifunctional ligands has emerged as a valuable strategy in the treatment of chronic pain. More precisely, µ-opioid pharmacophores were covalently combined to a series of antagonists targeting G protein-coupled receptors (here, the NK2 and NK3 receptors) known to cooperatively act with the opioid system or more widely described as nociception modulators. Following this approach, we previously reported the design of opioid agonist–NK1 antagonist hybrids, composed out of the putative opioid pharmacophore KGOP01 H-Dmt-d-Arg-Aba-βAla-NH_2_. As a second benchmark MOR ligand, the Dmt-DALDA pharmacophore was included, since this reference compound has been described as a highly µ-selective agonist with a favorable amphipathic profile for BBB crossing and improved metabolic stability [31,32]. The design of the constrained analogue KGOP01 addressed the beneficial effect of a concomitant activation of DOR for application in chronic pain treatment [28,33,34]. Because the opioid pharmacophores are primarily recognized through the *N*-terminus, they were covalently linked to a selection of NK2 and NK3 ligands through their *C*-terminus. The NK2 and NK3 ligands were selected based on their structure, featuring either a peptidic or a non-peptidic scaffold, and depending on a good biological affinity, selectivity, and activity. Such a differentiated nature of the NK pharmacophores could eventually lead to distinct pharmacokinetic properties. 

The Menarini Lab was a pioneer in the development of NK receptor ligands and the initial efforts were logically directed toward sequential modifications of the endogenous neurokinins SP, NKA, and NKB. In the first generation of patented NK2 receptor antagonists, the peptidic MEN 10,376 resulted from a d-tryptophan insertion in the truncated endogenous NKA(4-10) sequence and exhibited selectivity and nanomolar affinity toward NK2 [25]. Given this selectivity profile, it constituted an adequate peptidic neurokinin pharmacophore in the context of opioid-NK ligands. To answer metabolic issues, peptide scaffolds were then gradually modified into pseudo-peptidic drugs, and a new generation of NK antagonists was disclosed. Among those, MEN 15,596 or Ibodutant stood out as a NK2 antagonist with great potential for the treatment of abdominal pain [35,36], which ended up in clinical trials for IBS (Irritable Bowel Syndrome) therapy. Its parent compound, MEN 14,268, which mainly differs at the *C*-terminal basic appendage, displayed slightly reduced antagonism and NK2 affinity, but higher apparent permeability [13]. Considering synthetic feasibility, MEN 14,268 appeared more attractive. For the purpose of the current study, MEN 14,268 was eventually simplified by replacing the benzothiophene unit with a simple benzylamine group, leading to a pseudo-peptidic NK2 antagonist pharmacophore **SBL-OPNK-2** (Figure 1). Finally, as a representative of a non-peptidic scaffold, Talnetant matched, as this compound combines good affinity (nanomolar range), selectivity, and antagonist activity of the NK3 receptor [13]. This small molecule, bearing a central quinoline core, exhibited great potential in inhibiting nociception associated with intestinal distension [37]. To avoid any potent steric hindrance between the opioid and the NK3 pharmacophore **SBL-OPNK-3**, it was decided to insert a short ether linker in place of the hydroxyl group on the quinoline ring (Figure 1 and Scheme 2).

Synthesis of all pharmacophores and hybrids proceeded smoothly following adapted reported procedures, allowing access to an adequate amount of material for biological evaluation. 

In vitro binding studies demonstrated that the conjugation of the NK2 and NK3 pharmacophores generally allows for the selectivity toward MOR and DOR originally acquired by the individual parent opioid pharmacophores to be maintained, while still being much less active on KOR. In the same way as their opioid parent Dmt-DALDA, the three hybrids **SBL-OPNK-4**, **SBL-OPNK-6**, and **SBL-OPNK-8** all exhibited selectivity toward MOR, while unfortunately, the affinity and potency were reduced. In our hands, fusion of the Dmt-DALDA sequence with additional pharmacophores at the opioid’s *C*-terminal end generally led to such a decrease in affinity and potency (unpublished results). On the other hand, the KGOP01-based hybrids, **SBL-OPNK-5**, **SBL-OPNK-7**, and **SBL-OPNK-9**, all displayed both good affinity and activity toward MOR and DOR, despite slightly lowered *K*_i_ and EC_50_ values; results that can be correlated to the fusion of an additional pharmacophore. It could, therefore, be suggested that the impact of NK2 and NK3 pharmacophores on opioid activity will strongly depend on the nature of the opioid pharmacophore. The data suggest that anchorage of any *C*-terminal appendage to the constrained structure of KGOP01 is more tolerated, which is in line with previous studies [19,38,39]. In light of the promising opioid data, the KGOP01-based hybrids were therefore selected for further biological evaluation, and NK2 and NK3 receptor binding assays were performed. NK pharmacophores **SBL-OPNK-1** and **SBL-OPNK-3** both displayed similar affinity and potency as reported in the literature, with the important side note that compound **SBL-OPNK-3** represents an altered form of the literature compound (see Figure 1). Compound **SBL-OPNK-2**, however, failed to reach sufficient effects at the highest test concentrations. This result could be correlated to the development history of the FDA-approved Ibodutant and MEN 14,268, for which structural examination of the ‘*N*-terminal’ fragment was subjected to extensive efforts, underlining its critical impact on neurokinin efficacy [26]. Unlike the original benzothiophene moiety, the *N*-acetylbenzylamine group thus appeared detrimental for the activity and potency of **SBL-OPNK-2** on NK2. It was therefore highly satisfying to note that this negative effect disappeared for the corresponding NK2 hybrid **SBL-OPNK-7**, which displayed the best biological profile with subnanomolar affinity and activity. The latter compound even outperformed hybrid **SBL-OPNK-5** in terms of binding. It might be hypothesized that the KGOP01 pharmacophore adds extra key and stabilizing contacts with the neurokinin receptor binding site, a finding which was also previously noted for the opioid-neurokinin 1 receptor hybrid, SBCHM01 [28]. Structural analysis might be implemented in future studies to confirm this, but the observation of enhanced and (sub)nanomolar *IC*_50_ and *K*_i_ values, demonstrated for NK2 hybrid **SBL-OPNK-5** and NK3 hybrid **SBL-OPNK-9,** also supports this hypothesis. It can be noted that the increase in affinity was slightly less important for **SBL-OPNK-9**, suggesting that the binding ability of the more compact NK3 moiety is less influenced by the opioid unit. 

In this study, we disclosed, for the first time, a series of novel opioid agonist–non-opioid NK2 and NK3 receptor antagonist hybrid compounds as an unprecedented, yet promising approach toward innovative pain therapeutics. The NK2 and NK3 receptors have been overlooked and might provide beneficial effects compared to opioid-NK1 receptor ligands. As for any new multifunctional drugs, the challenge of conserving affinity and activity to both or more targets had to first be resolved, and the herein disclosed hybrids have eventually fulfilled this criterion. The covalent combination of neurokinin pharmacophores to the opioid agonist moiety globally improves neurokinin binding affinity, while maintaining low nanomolar µ and δ-opioid affinity and functional activity. These highly promising results will lead to further in vivo evaluation, which will be reported in due time.

## 4. Materials and Methods

### 4.1. Chemistry

#### Peptide Synthesis

Peptide compounds KGOP01, **SBL-OPNK-4**, and **SBL-OPNK-5** were assembled on Rink Amide resin (ChemImpex, Wood Dale, IL, USA—Loading 0.47 mmol/g) following iterative couplings of the required Fmoc-protected residues. Canonical l-amino acids (four equivalents with respect to the resin) were coupled in 30 min using a mixture of four equivalents HBTU/DIPEA in DMF. d-amino acids (2 equivalents with respect to the resin) were coupled for 1 h using a mixture of two equivalents HBTU/DIPEA in DMF. Fmoc-Aba-βAla-OH (1 equivalent), previously prepared according to the reported procedure [28], was coupled overnight using HBTU/DIPEA (one equivalent). Ultimately, 1.5 equivalents of Boc-Dmt-OH (commercially available) was coupled at the *C*-terminal position using DIC/Oxyma (1.5/3 equivalents) and stirred overnight. Full cleavage from the resin was carried out in standard conditions using a cocktail cleavage of TFA/TIS/H_2_O (95:2.5:2.5, *v*/*v*/*v*). Fully protected opioid pharmacophores Boc-Dmt-d-Arg(Pbf)-Phe-Lys(Boc)-OH **1** and Boc-Dmt-d-Arg(Pbf)-Aba-βAla-OH **2** were assembled on 2-chlorotrityl resin following standard conditions as previously described for Rink Amide resin procedures. Cleavage from the resin was carried out using HFIP/DCM (1:4, *v*/*v*), in order to preserve protecting groups for further coupling (detailed data available in the Supplementary Materials).

### 4.2. In Vitro Biological Evaluation

#### 4.2.1. Drugs

Cell culture media and fetal bovine serum were obtained from Euroclone (Pero, Italy) and supplements were purchased from Invitrogen (Paisley, UK). Standard ligands (dermorphin, dynorphin A, and DPDPE) were from Sigma-Aldrich Chemical Co. (Poole, UK) and were of the highest purity available. The radioligands: [^3^H]DAMGO, [^3^H][Ile^5,6^]deltorphin-2, and [^3^H]nor-binaltorphimine ([^3^H]nor-BNI) were from Perkin Elmer (Massachusetts, USA). Tested compounds: KGOP01, Dmt-DALDA, **SBL-OPNK-4**, **SBL-OPNK-5**, **SBL-OPNK-6**, **SBL-OPNK-7**, **SBL-OPNK-8**, and **SBL-OPNK-9**. 

#### 4.2.2. Opioid Receptor Radioligand Binding Assay 

Binding affinities for opioid receptors (MOR, DOR, KOR) were determined by displacing [^3^H]-labeled, commercially available ligands from a commercially available cell membrane preparation from Chinese hamster ovary (CHO) cells expressing human recombinant receptors of interest (Perkin Elmer).

The following radioligands were used: [^3^H]DAMGO for MOR, [^3^H][Ile^5,6^]deltorphin-2 for DOR, and [^3^H]nor-binaltorphimine ([^3^H]nor-BNI) for KOR. 

Membrane preparations were incubated at 25 °C for 120 min with an appropriate concentration of a tested compound in the presence of 0.5 nM radioligand in a total volume of 0.5 mL of Tris/HCl (50 mM, pH 7.4), containing bovine serum albumin (BSA, 1 mg/mL), bacitracin (50 mg/L), bestatin (30 µM), and captopril (10 µM). Non-specific binding was determined in the presence of 10 µM of naloxone. Incubations were terminated by rapid filtration through Whatman GF/B (Brentford, UK) glass fiber strips, which were pre-soaked for 2 h in 0.5 % polyethylamine using Millipore Sampling Manifold (Billerica, MA, USA). The filters were washed three times with 4 mL of ice-cold Tris buffer solution. The bound radioactivity was measured in Packard Tri-Carb 2100 TR liquid scintillation counter (Ramsey, MN, USA) after overnight extraction of the filters in 4 mL of Perkin Elmer Ultima Gold scintillation fluid (Wellesley, MA, USA). Three independent experiments for each assay were carried out in duplicate. 

The data were analyzed by a nonlinear least square regression analysis computer program Graph Pad PRISM 6.0 (GraphPad Software Inc., San Diego, CA, USA). The *IC*_50_ values were determined from the logarithmic concentration–displacement curves, and the values of the inhibitory constants (*K*_i_) were calculated according to the equation of Cheng et al. [40]. 

#### 4.2.3. Calcium Mobilization Assay

CHO cells stably co-expressing human recombinant MOR or KOR opioid receptors and the *C*-terminally modified Gα_qi5_, and CHO cells co-expressing the human recombinant DOR opioid receptor and the Gα_qG66Di5_ chimeric protein were a generous gift from Prof. Girolamo Calo’, Ferrara University, Italy and have been successfully used in our laboratory [30]. 

The tested compounds were dissolved in 5% DMSO in bi-distilled water to the final concentration of 1 mM. The successive dilutions were made in the HBSS/HEPES (20 mM) buffer (containing 0.005% BSA fraction V).

For the experiment, cells were seeded at a density of 50,000 cells/well into 96-well black, clear-bottom plates. After 24 h incubation, the cells were treated with the loading solution of the culture medium supplemented with 2.5 mM probenecid, 3 µM of the calcium-sensitive fluorescent dye Fluo-4 AM, and 0.01% pluronic acid for 30 min at 37 °C. The loading solution was aspirated and a 100 µL/well of the assay buffer (Hank’s Balanced Salt Solution (HBSS) supplemented with 20 mM HEPES, 2.5 mM probenecid, and 500 µM Brilliant Black) was added.

After placing both plates (cell culture and compound plate) into the FlexStation III plate reader, the on-line additions were carried out in a volume of 50 µL/well and fluorescence changes were measured at 37 °C.

Agonist potencies were given as EC_50_ representing the molar concentration of an agonist that produces 50% of the maximal possible effect. Concentration–response curves were fitted with the four parameters logistic nonlinear regression model:

$$\mathrm{Effect}=\mathrm{baseline}+\frac{E_{\max}-\mathrm{baseline}}{1+10^{{(\mathrm{logEC}}_{50}-X)\cdot n}}$$

where X is the agonist concentration and n is the Hill coefficient. Ligand efficacy was expressed as intrinsic activity (α) calculated as the E_max_ of the ligand to E_max_ of the standard agonist ratio.

At least five independent experiments for each assay were carried out in duplicate. Curve fittings were performed using Graph Pad PRISM 6.0 (GraphPad Software Inc., San Diego, CA, USA). Data were statistically analyzed with one way ANOVA followed by the Dunnett’s test for multiple comparisons; *p* values less than 0.05 were considered significant.

#### 4.2.4. NK2 and NK3 Binding Assays

Binding affinities for neurokinin receptors, NK2 and NK3, were carried out through Eurofins’ service according to standard procedures, in which affinities were determined by displacing [^125^I]-labelled NKA and [^3^H]-labeled SR 142801 (Table 4) [41,42].

The *IC*_50_ values (concentration causing a half-maximal inhibition of control specific binding) and Hill coefficients (nH) were determined by non-linear regression analysis of the competition curves generated with mean replicate values using Hill equation curve fitting:

$$Y=D+\left(\frac{A-D}{1+{\left(\frac{C}{C_{50}}\right)}^{nH}}\right)$$

where *Y* = specific binding; *A* = left asymptote of the curve; *D* = right asymptote of the curve; *C* = compound concentration; *C*_50_ = *IC*_50_; and *nH* = slope factor. This analysis was performed using software developed at Cerep (Hill software) and validated by comparison with data generated by the commercial software SigmaPlot^®^ 4.0 for Windows^®^ (© 2021 by SPSS Inc., Chicago, IL, USA).

The inhibition constants (*K*_i_) were calculated using the Cheng Prusoff equation

$$K_{i}=\frac{IC_{50}}{\left(1+\frac{L}{K_{D}}\right)}$$

where *L* = concentration of radioligand in the assay; and *K_D_* = affinity of the radioligand for the receptor. A Scatchard plot is used to determine the *K_D_*.

## Figures and Tables

**Table 1:** Binding affinity of opioid receptors of the tested compounds.

Compound	*K*_i_ [nM] ± SEM		
	MOR	DOR	KOR
Dmt-DALDA	0.61 ± 0.02	2313	128.98 ± 8.02
KGOP01	0.12 ± 0.06	4.88 ± 0.11	72.75 ± 4.32
**SBL-OPNK-4**	1.02 ± 0.03	1251.18	90.50 ± 5.69
**SBL-OPNK-5**	2.11 ± 0.04	10.02 ± 3.20	90.72 ± 0.94
**SBL-OPNK-6**	74.93 ± 0.15	22377	100.44 ± 1.16
**SBL-OPNK-7**	2.83 ± 0.02	3.26 ± 0.09	52.98 ± 3.20
**SBL-OPNK-8**	119.68 ± 5.25	4574.17	103.83 ± 6.12
**SBL-OPNK-9**	5.43 ± 0.08	6.57 ± 4.02	97.15 ± 4.72

**Table 2:** Effects of reference agonists and tested compounds at human recombinant opioid receptors coupled with calcium signaling via chimeric G proteins.

	MOR		DOR		KOR	
	EC_50_ ± SEM (nM)	α ± SEM	EC_50_ ± SEM (nM)	α ± SEM	EC_50_ ± SEM (nM)	α ± SEM
dermorphin	4.64 ± 0.57	1.00	Inactive ^a^		inactive	
DPDPE	inactive		37.62 ± 15.80	1.00	inactive	
dynorphin A	230 ± 54 ^a^	0.83 ± 0.10 ^a^	19.70 ± 0.82 ^a^	0.99 ± 0.04 ^a^	4.76 ± 1.40	1.00
Dmt-DALDA	5.61 ± 0.61	1.00 ± 0.06	inactive		3179 ± 1583	0.55 ± 0.02
KGOP01	0.54 ± 0.06	1.04 ± 0.04	75.61 ± 13.09	1.02 ± 0.07	crc incomplete	
**SBL-OPNK-4**	815 ± 145	0.74 ± 0.05	inactive		crc incomplete	
**SBL-OPNK-5**	10.21 ± 0.67	0.91 ± 0.04	117.73 ± 25.96	1.01 ± 0.03	415 ± 133	0.44 ± 0.06
**SBL-OPNK-6**	988 ± 82	0.67 ± 0.05	inactive		crc incomplete	
**SBL-OPNK-7**	13.08 ± 1.84	0.89 ± 0.04	66.53 ± 11.65	1.02 ± 0.02	inactive	
**SBL-OPNK-8**	916 ± 106	0.65 ± 0.02	inactive		crc incomplete	
**SBL-OPNK-9**	31.30 ± 7.39	0.83 ± 0.05	84.07 ± 17.95	1.02 ± 0.03	crc incomplete	

^a^ Data from reported literature [30]. Maximum tested concentration: 10 µM.

**Table 3:** Neurokinin receptor NK2 and NK3 ligand binding assay.

Compounds		*IC*_50_ [nM] ± SEM	*K*_i_ [nM] ± SEM
References	[Nle^10^]-NKA(4-10)	2.1	1.1
	SB 222200	10	5.7
NK2 pharmacophores	**SBL-OPNK-1**	33.6 ± 11.9 ^a^	18.3 ± 13.0
	**SBL-OPNK-2**	n.c. ^a^	n.c.
NK3 pharmacophore	**SBL-OPNK-3**	20.3 ± 7.2 ^b^	11.0 ± 7.8
Hybrids	**SBL-OPNK-5**	3.0 ± 1.0 ^a^	1.6 ± 1.1
	**SBL-OPNK-7**	0.63 ± 0.22 ^a^	0.34 ± 0.24
	**SBL-OPNK-9**	6.7 ± 2.4 ^b^	3.6 ± 2.5

^a^ Determined on the NK2 receptor. ^b^ Determined on the NK3 receptor. n.c., not calculated: concentration–response curve showed less than 25% inhibitory binding at the highest validated test concentrations.

**Table 4:** NK2 and NK3 binding affinities assay conditions.

Receptors	Source	Ligand	Conc.	K_D_	Non-Specific	Incubation	Detection Method
NK2 (h) (agonist radioligand)	Human recombinant (CHO cells)	[^125^I]NKA	0.1 nM	0.12 nM	[Nleu10]-NKA(4-10) (300 nM)	60 min RT	Scintillation counting
NK3 (h) (antagonist radioligand)	Human recombinant (CHO cells)	[^3^H]SR 142801	0.4 nM	0.47 nM	SB 222200 (10 μM)	120 min RT	Scintillation counting

## Supplementary Materials

The following are available online, Scheme S1: Solid-phase peptide and peptidomimetic synthesis; Scheme S2: Solution-phase peptides and peptidomimetics synthetic pathways; Scheme S3: NK3 pharmacophore and hybrids synthetic pathway; Figure S1: Correlation between radioligand binding and calcium mobilization assays for MOR and DOR.

## Sample Availability

Samples of compounds are not available from the authors.
