# Supplementary material for: Harnessing the Anti-Nociceptive Potential of NK2 and NK3 Ligands in the Design of New Multifunctional μ/δ-Opioid Agonist–Neurokinin Antagonist Peptidomimetics
Source: Molecules. 2021 Sep 6;26(17):5406. doi: 10.3390/molecules26175406 (PMC8434392; doi:10.3390/molecules26175406)
Supplement: Supplementary file 1 [file molecules-26-05406-s001.zip › molecules-1350988-supplementary.pdf]

Harnessing NK2 and NK3 ligands anti-nociceptive potential in the design of new multifunctional μ/δ-opioid agonist-neurokinin antagonist peptidomimetics

Charlène Gadais,[1,2]* Justyna Piekielna-Ciesielska,[3] Jolien De Neve,[1] Charlotte Martin,[1] Anna Janecka,[3] Steven Ballet[1]*

[1] Research Group of Organic Chemistry, Departments of Bioengineering Sciences and Chemistry, Vrije Universiteit Brussel, Pleinlaan 2, 1050 Brussels, Belgium

[2] Institut des Sciences Chimiques de Rennes, Equipe CORINT, UMR 6226, Université de Rennes 1, 2 avenue du Pr. Léon Bernard, 35043 Rennes Cedex, France

[3] Department of Biomolecular Chemistry, Faculty of Medicine, Medical University of Lodz, 92-215 Lodz, Poland

Correspondence: steven.ballet@vub.be (S.B.) ch.gadais@gmail.com (C.G.);

Table of content

1. Characterization table 3

2. General analytical and semi-preparative methods 4

3. Synthetic procedures 5

3.1. Solid-phase peptide and peptidomimetic synthesis procedures – access to NK2 pharmacophores and hybrids 5

3.2. Solution-phase peptide and peptidomimetic synthesis procedures – access to NK2 pharmacophores and hybrids 7

3.3. NK3 pharmacophore and hybrid synthesis procedures 11

4. NMR spectra and HPLC chromatographs 13

5. Correlation between radioligand binding and calcium mobilization assays for MOR and DOR 22

1. Characterization table

Table S1

Ref cpd	Sequence/structure	Formula	MW (g/mol^{-1})	Calc.[M+H]$^+$	HRMS found [M+H]$^+$	HPLC t_R (min)
**Dmt-DALDA**	H-Dmt-D-Arg-Phe-Lys-OH	$C_{32}H_{48}N_8O_6$	982.8 (3 TFA)	641.3770	641.3759	2.15 [1] / 1.42 [2]
**KGOP01**	H-Dmt-D-Arg-Aba-βAla-NH_2	$C_{30}H_{42}N_8O_5$	822.8 (2 TFA)	595.3356	595.3162	2.33 [1]
**SBL-OPNK-1**	Ac-Asp-Tyr-D-Trp-Val-D-Trp-D-Trp-Lys-NH_2	$C_{59}H_{70}N_{12}O_{11}$	1237.3 (1 TFA)	1123.5360	1123.5322	2.33 [2]
**SBL-OPNK-2**	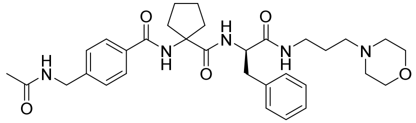	$C_{32}H_{45}N_5O_5$	691.7 (1 TFA)	578.3372	578.3330	2.71 [1] / 16.69 [3]
**SBL-OPNK-3**	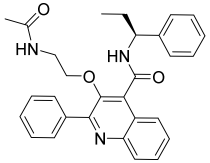	$C_{29}H_{29}N_3O_3$	467,6 (0 TFA)	468.2287	468.2224	3.42 [1] / 18.24 [3]
**SBL-OPNK-4**	H-Dmt-D-Arg-Phe-Lys-Asp-Tyr-D-Trp-Val-D-Trp-D-Trp-Lys-NH_2	$C_{89}H_{114}N_{20}O_{15}$	2160.0 (4 TFA)	1703.8851	1703.8905	3.13 [1]
**SBL-OPNK-5**	H-Dmt-D-Arg-Aba-βAla-Asp-Tyr-D-Trp-Val-D-Trp-D-Trp-Lys-NH_2	$C_{87}H_{107}N_{19}O_{15}$	2000.9 (3 TFA)	1658.8267	1658.8282	2.28 [2]
**SBL-OPNK-6**	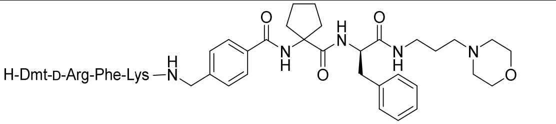	$C_{62}H_{87}N_{13}O_9$	1500,5 (3 TFA)	1158.6828	1158.6848	2.92 [1]
**SBL-OPNK-7**	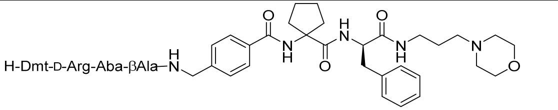	$C_{60}H_{80}N_{12}O_9$	1341.4 (2 TFA)	1113.6250	1113.6221	2.88 [1]
**SBL-OPNK-8**	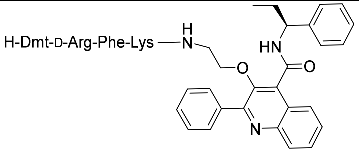	$C_{59}H_{73}N_{11}O_7$	1390.34 (3 TFA)	1048.5773	1048.5756	3.25 [1]
**SBL-OPNK-9**	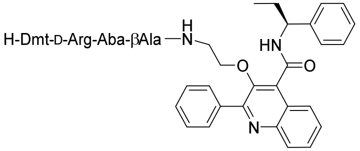	$C_{57}H_{66}N_{10}O_7$	1231.2 (2 TFA)	1003.5194	1003.5134	3.42 [1]

Dmt: 2',6' dimethyl tyrosine

2. General analytical and semi-preparative methods

NMR

A Bruker Avance 300 spectrometer was used at 300 MHz (^{1}H NMR) and 75 MHz (^{13}C NMR) at room temperature. The chemical shifts were reported in delta (δ) units in parts per million (ppm) relative to the signal of the deuterated solvent. For $CDCl_3$, the singlet in ^{1}H NMR was calibrated at 7.27 ppm and the central line of the triplet in ^{13}C NMR at 77.0 ppm. When recording in MeOD or DMSO-d_6, the calibration was performed at 3.31 ppm and 2.50 ppm for ^{1}H NMR and 49.0 ppm and 39.5 ppm for ^{13}C NMR, respectively. Assignments were made using one dimensional (1D) ^{1}H and ^{13}C spectra. Multiplicities were described as singlet (s), doublet (d), triplet (t), quartet (q), multiplet (m), broad (br), or a combination thereof. The corresponding coupling constants (*J* values) were reported in Hertz (Hz).

HPLC

Analytical RP-HPLC was performed on a VWR-Hitachi Chromaster HPLC with a Chromolith HighResolution RP-18C column from Merck (150 mm x 4.6 mm, 1.1 µm, 150 Å). The flow rate was 3 ml/min, and UV detection was done at 214 nm. The solvent system used consisted of 0.1% TFA in ultrapure water (A) and 0.1% TFA in acetonitrile (B) with a gradient from 3% B to 100% B over 6 minutes.

GC-MS

GCMS analysis was performed on an Agilent GC unit 7820A, equipped with a HP-5MS column (30 m, 0.250 mm of diameter, 0.25 µm film layer), and coupled to a MS 5975 unit. The general analysis method was performed following a gradient from 50 °C to 325 °C with 16°C/min.

LC-MS

For LC-MS analysis, two systems were used. *System A*: the HPLC unit used was a Waters 600 system combined with a Waters 2487 UV detector at 215 nm and as stationary phase a Vydac MS RP C18-column (150 mm x 2.1 mm, 3 µm, 300 Å). The solvent system used was 0.1 % formic acid in water (A) and 0.1 % formic acid in acetonitrile (B) with a gradient going from 3 % to 100 % B over 20 minutes with a flow rate of 0.3 ml/min. The MS unit, coupled to the HPLC system, was a Micromass QTOF-micro system. *System B*: HPLC system—Diode Array Detector (LC-DAD) (Shimadzu, Marne La Vallée, France) and a mass spectrometer with a single quadrupole mass analyzer (Advion expression CMS). Ionization was performed by electrospray in negative or positive mode (ESI) for low resolution analysis. A Prevail C18 column (5 µm, 250 × 4.6 mm, hypersil gold, Thermofischer), was used for HPLC, and a gradient system was applied: A (0.1% formic acid in water) and B (0.1% formic acid in acetonitrile). The following gradient was applied at a flow rate of 0.8 ml/min in the HPLC system: initial: 100% (A); from 0 to 5 minutes: 100% (A); from 5 to 25 minutes: 100% (A)/0% (B) to 0% (A)/100% (B); from 25 to 35 minutes: 100% B; from 35 to 40 minutes: 100% (A)/0% (B) to 0% (A)/100% (B); from 40 to 45 minutes: 100% (A). A 0.2 ml/min split was applied for mass spectrometry.

HRMS

The high-resolution mass spectroscopy was performed on LCMS *system A* (as described above). The same MS system was used with reserpine (2.10-3 mg/ml) solution in water:acetonitrile (1:1) as reference.

Semi-preparative HPLC

Semi-preparative RP-HPLC-purifications were done using a Gilson HPLC system with Gilson 322 pump equipped with a Grace® Vydac 150HC C18 (250 mm x 22 mm, 10 µm) column and Waters UV/VIS-156 detector at 215 nm (*Prep1*), or using a Knauer HPLC system equipped with a RP-18C ReproSil-Pur ODS-3 column (150 mm x 16 mm, 5 µm) (*Prep2*), or alternatively, peptides were purified by RP-HPLC on an XBridge (BEH130 Prep C18) semi-prep column (19 × 150 mm; 5 µm) in a Waters 600 system (*Prep3*). The same solvent system was used as applied for the analytical RP-HPLC with a flow rate of 10 or 20 ml/min.

3. Synthetic procedures

Unless stated otherwise, all commercial chemicals were used without further purification. Non-commercial starting materials were prepared based on literature procedures and are described below.

3.1. Solid-phase peptide and peptidomimetic synthesis procedures – access to NK2 pharmacophores and hybrids

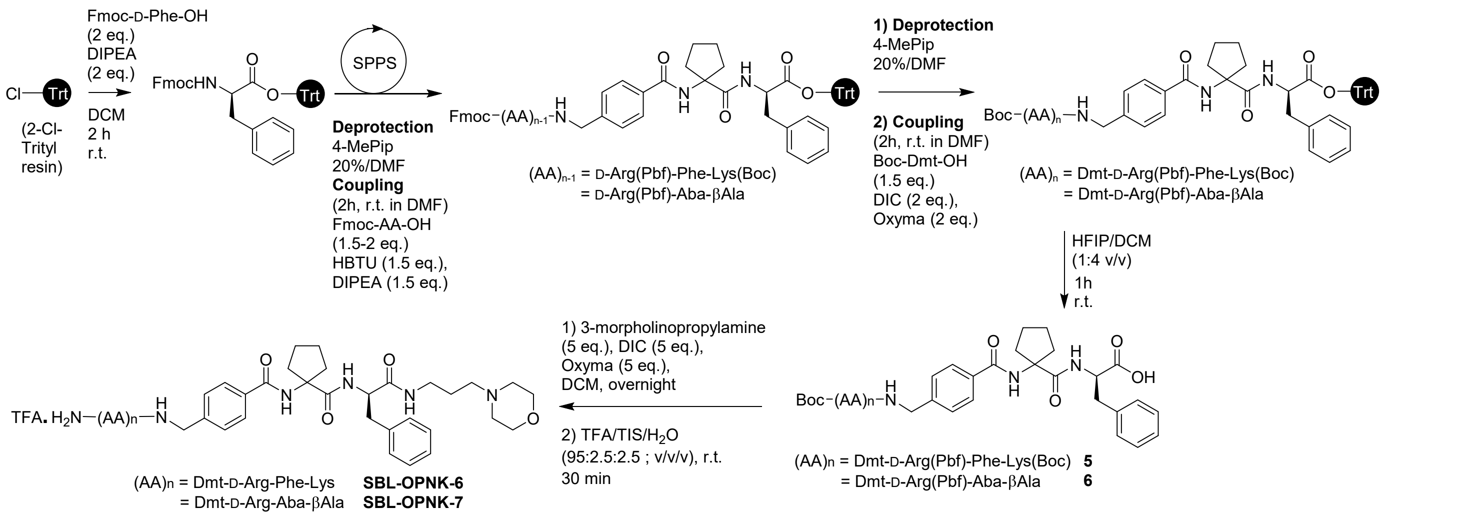

Scheme S1 Solid-phase peptide and peptidomimetic synthesis

Following standard peptide synthesis procedures, peptide-based pharmacophores **Dmt-DALDA**, **KGOP01** and **SBL-OPNK-1**, and peptide-based hybrids **SBL-OPNK-4** and **SBL-OPNK-5** were synthesized on 0.1 mmol scale, on rink amide resin (0.47 mmol/g), and hybrid compounds **SBL-OPNK-6** and **SBL-OPNK-7** were partly synthesized on 2-chlorotrityl resin (0.78 mmol/g), using propylene reactor vessel. Deprotection and coupling steps were carried out according to the conditions described in the following table.

1	Swelling of the resin in DMF	2 x 5 min
2	Deprotection with 20% 4-MePip in DMF	1 x 5 min
3	Deprotection with 20% 4-MePip/DMF	1 x 15 min
4	Washing with DMF	3 x 1 min
5	Washing with DCM	3 x 1 min
6	Coupling[a]	1 x 45 min
7	Washing with DMF	3 x 1 min
8	Washing with DCM	3 x 1 min
9	Kaiser test	
10	(Small scale cleavage)[b]	
11	N-terminal acetylation[c]	1 x 30 min

[a] Coupling conditions on 0.1 mmol scale: see below
[b] Every addition of 2 or 3 residues
[c] N-terminal acetylation after Fmoc deprotection

Swelling: Prior to the synthesis, the resin was swelled for 5 minutes in DMF on a shaker. Solvent was filtered and the swelling was repeated a second time.

Deprotection: Fmoc deprotection was carried out, 2 times, using 20% 4-methylpiperidine (4-MePip) in DMF (v/v) for 5 and 15 minutes, respectively.

Amino acid coupling: Fmoc-protected amino acid (0.3 mmol, 3 equiv.), HBTU (0.3 mmol, 114 mg, 3 equiv.), and DIPEA (0.3 mmol, 69 µL, 3 equiv.) were solubilized in 2 ml DMF, and stirred for 5 minutes at room temperature before being transferred into the reactor. Single coupling was applied, except for arginine residues that were coupled twice. The coupling mixture was pre-activated for 5 minutes prior to its addition to the resin. In case of *N*-terminal coupling of Boc-Dmt-OH, DIC (2 equiv.) and Oxyma (2 equiv.) was used as a coupling reagent cocktail.

Kaiser test: The Kaiser test was performed after each coupling. If the test was positive (blue/purple beads/solution) the coupling was repeated.

The Kaiser test solutions:
Solution A: Phenol in EtOH (4:1) = 75 ml/80 g phenol + 20 ml EtOH.
Solution B: 0.28 M ninhydrin in EtOH (1 g ninhydrin in 20 ml in EtOH).
Solution C: 1 ml of KCN 0.01mM (aqueous) + 49 ml pyridine.

Few beads of resin was taken out and added to a test tube. 2 drops of each solution A, B and C were added, and the mixture was heated at 110 °C for 2 min, and the solution observed.

***N*-terminal acetylation step:** After Fmoc-deprotection of the *N*-terminal amino acid and washing steps, a mixture of freshly made acetic anhydride/pyridine 3:2 v/v was added to the resin and shaken for 30 minutes. Subsequently, the resin was washed with DCM (3x), isopropanol (3x), and the completion of the acetylation checked using the Kaiser test. If needed, the full operation is repeated a second time.

Cleavage and deprotection: The resin was washed carefully with DCM (3 x 1 min).

Cleavage of Rink amide resin: Freshly made cleavage mixture (TFA/TIS/H_2O, 95:2.5:2.5, v/v/v) was transferred to the resin. The suspended resin was shaken for 1 hour at room temperature, and the solution subsequently filtered off and carefully collected in a round-bottom flask. Th resin was washed with DCM and the filtrate was concentrated *in vacuo*. Crude peptide was precipitated in cold diethylether before being purified by mean of semi-preparative HPLC.

Cleavage of 2-chlorotrityl resin: To maintain protecting groups, cleavage was carried out using a freshly made cleavage cocktail: HFIP/DCM (1:4 v/v). The suspended resin was shaken for 1 hour at room temperature, after which collection of the cleaved peptide is carried out in the same way as for rink amide resin cleavage. The resin was thoroughly washed with DCM and the filtrate concentrated *in vacuo*. Crude peptide was precipitated in cold diethylether before being purified using semi-preparative HPLC.

3.2. Solution-phase peptide and peptidomimetic synthesis procedures – access to NK2 pharmacophores and hybrids

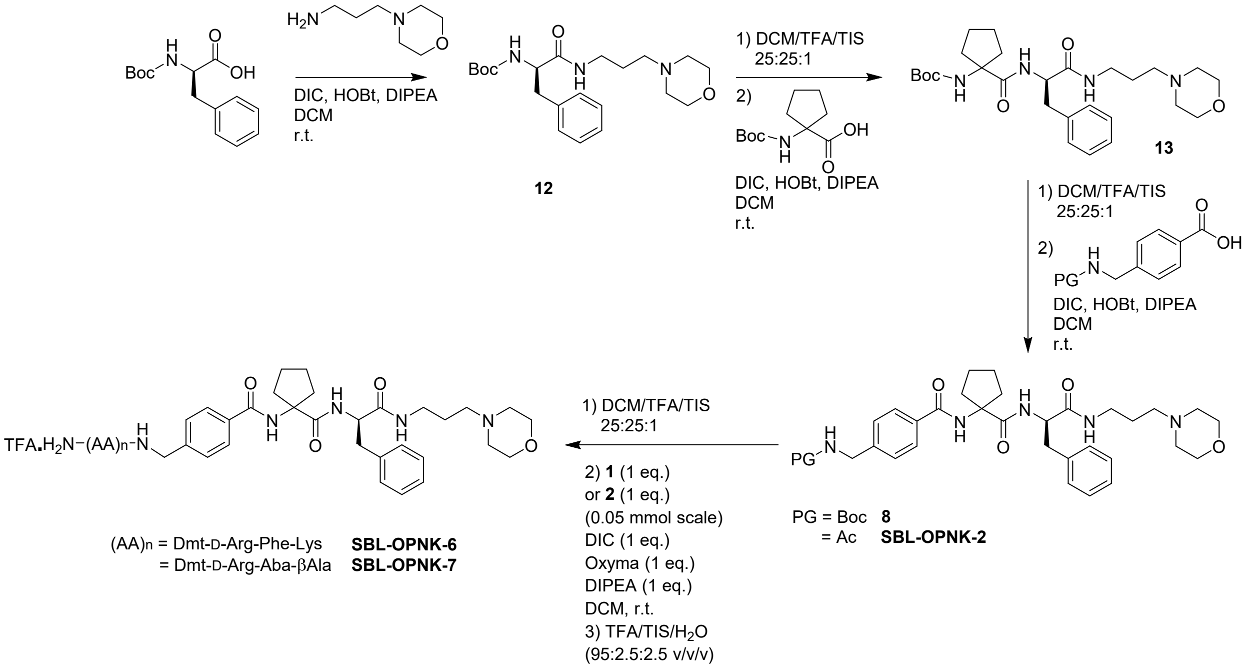

Scheme S2 Solution-phase peptides and peptidomimetics synthetic pathways

General coupling procedure A

Boc-protected amino acid (1 equiv.) was suspended in dry DCM (10 ml/2 mmol), and cooled down to 0°C. HOBt (1.1 equiv.) and DIC (1.1 equiv.) were added at 0°C, and the resulting mixture was stirred for 5-10 minutes. To this mixture, a solution of the appropriate amine (1 equiv.) in 5 ml of DCM was then slowly added, followed by DIPEA (1.1 equiv.). The reacting mixture was then warmed up to room temperature and stirred overnight until completion. The reaction was monitored by TLC plate with DCM/MeOH. DCM was evaporated to dryness and residue was then triturated into AcOEt. The urea was filtered off and the filtrate was recovered and washed. The resulting layers were separated and the organic layer was washed by $NaHCO_3$ 10% (2x) and brine, dried over $MgSO_4$ and concentrated under vacuo to give the title compound in sufficient purity to proceed with following steps.

General Boc-deprotection procedure B

Boc-protected amine compound was treated at room temperature by a mixture of DCM/TFA/TIS in 25:25:1 ratio. The mixture was stirred for 1-3 hours at room temperature and, then, concentrated to dryness. Crude product was triturated with cold diethyl ether. Overnight freezing can be used to help complete precipitation. Organic supernatant was eliminated and residue was triturated a second time with cold diethyl ether. Precipitate was filtered, rinsed with cold diethyl ether and dried or lyophilized to give the title compound as TFA salt, which was directly engaged in following coupling.

Amine-protection steps of starting building blocks

∇ **Synthesis of *N*-Acetyl-aminomethylbenzoic acid 3**

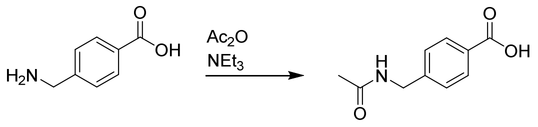

3

4-aminomethyl benzoic acid (0.75 g, 5 mmol, 1 equiv.) was dissolved in 15 mL dry THF, acetic anhydride (4.7 mL, 50 mmol, 10 equiv.) was then added at 0° C, followed by triethylamine (0.77 mL, 5.5 mmol, 1.1 equiv.). The mixture which was stirred at room temperature overnight. After reaction completion, solvent was evaporated and crude product solution was acidified with HCl 1 N until precipitation. Solid was filtered and rinsed with cold HCl 1M. Crude product was recrystallized from HCl 1 N to give the title compound **3** in 78% yield.

IUPAC-name: 4-(acetamidomethyl)benzoic acid **3**; **Appearance**: white solid; **Molecular formula**: $C_{10}H_{11}NO_3$; **Molecular weight**: 193.20 g.mol^{-1}; **Yield**: 78% (0.75 g); **^{1}H-NMR** (300 MHz, MeOD): 8.00 (d, *J* = 8.4 Hz, 2H), 7.40 (d, 8.5 Hz, 2H), 4.44 (s, 2H), 2.03 (s, 3H); **^{13}C-NMR** (75 MHz, MeOD): 173.3, 169.6, 145.4, 131.0, 130.8, 1284, 43.9, 22.4, **GC-MS** (EI, 70 eV) m/z (%) calcd. for $C_{10}H_{11}NO_3$ [M]$^{+}$: 193.07, found: 193.1 (M^{+}), 150, 106. Data are consistent with analytical data reported from commercial sources.

∇ **Synthesis of *N*-Boc-aminomethylbenzoic acid 4**

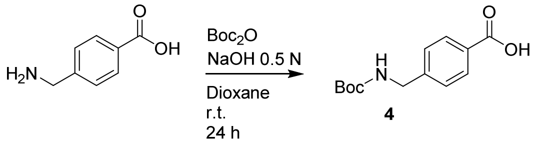

4

4-aminomethyl benzoic acid (0.75 g, 5 mmol, 1 equiv.) was dissolved in a mixture of 1,4-dioxane (15 mL) and NaOH 0.5 N (15 mL) at 0° C and di-*tert*-butyldicarbonate (1.20 g, 5.5 mmol, 1.1 equiv.) was added. Then the mixture was allowed to react at room temperature, under magnetic stirring for 24 hours. Then the reaction was stopped, concentrated to 1/3 volume and acidified to pH=2 with HCl 2 N. The obtained mixture was then extracted with ethyl acetate (3 x 10 mL), and the combined organic phases were dried over Na_2SO_4, filtered off and concentrated under reduced pressure, to give the desired intermediate **4** without further purification step in 86% yield.

IUPAC-name: 4-(((*tert*-butoxycarbonyl)amino)methyl)benzoic acid **4**; **Appearance**: white solid; **Molecular formula**: $C_{13}H_{17}NO_4$; **Molecular weight**: 251,28 g.mol^{-1}; **Yield**: 86% (g); **^{1}H-NMR** (300 MHz, MeOD): 7.99 (d, *J* = 8.3 Hz, 2H), 7.38 (d, *J* = 8.3 Hz, 2H), 4.31 (s, 2H), 1.47 (s, 9H); **^{13}C-NMR** (75 MHz, MeOD): 169.7, 158.6, 146.5, 130.9, 130.6, 128.0, 80.3, 44.7, 28.7. **GC-MS** (EI) m/z calcd. for $C_{13}H_{17}NO_4$ [M]$^{+}$: 251.12, found: 150 (M-Boc), 105. Data are consistent with literature [1].

∇ **Synthesis of *N*-Boc-cycloleucine**

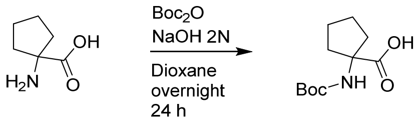

Cycloleucine (0.65 g, 5 mmol, 1 equiv.) was dissolved in 10 mL of 1,4-dioxane. To this solution, was added 2.5 mL of 2N NaOH and the solution was cooled in an ice-water bath. Di-*tert*-butyl-dicarbonate (1.20 g, 5.5 mmol, 1.1 equiv.) was added to the solution and the reaction mixture was stirred overnight at room temperature. Dioxane was then removed under reduced pressure and 50 mL of ethyl acetate was added to the remaining aqueous solution. The mixture was cooled in an ice-water bath and the pH of the aqueous layer was adjusted to about 3 by adding 4 N HCl. The organic layer was separated and the aqueous layer

was extracted once more with ethyl acetate. The two organic layers were combined, washed with water, dried over anhydrous $MgSO_4$, filtered, and concentrated to dryness under reduced pressure. The residue was triturated in ethyl acetate/hexanes to give the title product in 46% yield.

IUPAC-name: 1-((*tert*-butoxycarbonyl)amino)cyclopentane-1-carboxylic acid ; **Appearance**: white solid; **Molecular formula**: $C_{11}H_{19}NO_4$; **Molecular weight**: 229,28 g.mol^{-1}; **Yield**: 46% (0.53 g); **^{1}H-NMR** (300 MHz, MeOD): 2.31-2.14 (m, 2H), 1.96-1.91 (m, 2H), 1.79-1.76 (m, 4H), 1.44 (s, 9H); **GC-MS** (EI, 70 eV) m/z calcd. for $C_{11}H_{19}NO_4$ $[M]^{+*}$: 229.13, found: 128.1 (M-Boc), 84.1. Data consistent with literature [2].

NK2 intermediates synthesis and characterization

- **Synthesis of intermediate 12**

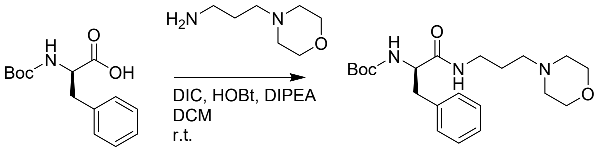

12

Boc-D-Phe-OH (2.12 g, 8 mmol, 1 equiv.) was coupled to 3-morpholinopropylamine (1.29 mL, 8.8 mmol, 1.1 equiv.) following general coupling procedure A. The crude product was chromatographed with DCM/MeOH (96:4) to give the title compound in 42% yield.

IUPAC-name: *tert*-butyl (*R*)-(1-((3-morpholinopropyl)amino)-1-oxo-3-phenylpropan-2-yl)carbamate **12**; **Appearance**: white solid; **Molecular formula**: $C_{21}H_{33}N_3O_4$; **Molecular weight**: 391,51 g.mol^{-1}; **Yield**: 42% (1.31 g); **^{1}H-NMR** (300 MHz, $CDCl_3$): 7.30-7.18 (m, 5H), 6.98 (s broad, 1H, NH), 5.07 (s broad, 1H, NH), 4.27-4.19 (m, 1H), 3.62-3.59 (m, 4H), 3.28-3.22 (m, 2H), 3.11-2.96 (m, 2H), 2.32-2.22 (m, 6H), 1.61-1.47 (m, 2H), 1.40 (s, 9H); **^{13}C-NMR** (75 MHz, $CDCl_3$): 170.8, 155.1, 136.8, 129.4, 128.5, 126.8, 80.0, 66.9, 57.7, 56.2, 53.6, 39.3, 39.0, 28.2, 24.4. **LC-MS** (ESI+) m/z calcd. for $C_{21}H_{33}N_3O_4$ $[M+H]^+$: 392.3, found : 392.3 , t_R = 12.96 min. (*system B*) Data are consistent with literature [3].

- **Synthesis of intermediate 13**

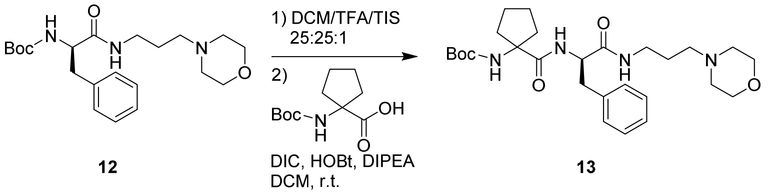

Following general procedure B, intermediate **12** (665 mg, 1.7 mmol, 1 equiv.) was first Boc-deprotected by treatment with DCM/TFA/TIS as described, and the resulting TFA salt was then coupled to Boc-protected cycloleucine following general procedure A. The crude product was chromatographed with DCM/MeOH (95:5) to give the title compound **13** in 82% yield.

IUPAC-name: *tert*-butyl (*R*)-(1-((1-((3-morpholinopropyl)amino)-1-oxo-3-phenylpropan-2-yl)carbamoyl)cyclopentyl)carbamate **13**; **Appearance**: white solid; **Molecular formula**: $C_{27}H_{42}N_4O_5$; **Molecular weight**: 502,66 g.mol^{-1}; **Yield**: 82% (700 mg); **^{1}H-NMR** (300 MHz, $CDCl_3$): 7.31-7.17 (m, 5H), 7.09 (m broad, 1H, NH), 6.56-6.53 (m broad, 1H, NH), 4.66-4.59 (m, 1H), 3.68-3.65 (m, 4H), 3.36-3.05 (m, 4H), 2.42-2.29 (m, 6H + NH), 2.00-1.88 (m, 2H), 1.80-1.56 (m, 8H), 1.34 (s, 9H); **^{13}C-NMR** (75 MHz, $CDCl_3$): 173.4, 170.7, 155.3, 136.8, 129.2, 128.6, 126.9, 80.7, 66.9, 56.4, 54.0, 53.5, 38.3, 38.1, 37.3, 35.9, 28.1, 25.5, 24.2. **LC-MS** (ESI+) m/z calcd. for $C_{27}H_{43}N_4O_5$ $[M+H]^+$: 503.3, found : 503.4 , t_R = 14.84 min (*System B*).

- **Synthesis of intermediate 8**

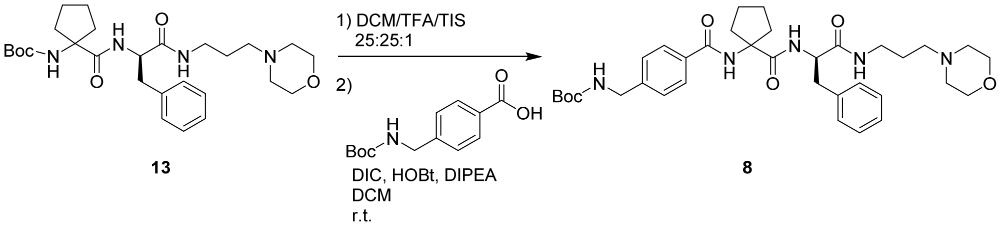

Following general procedure B, intermediate **13** (67 mg, 0.13 mmol, 1 equiv.) was first Boc-deprotected by treatment with DCM/TFA/TIS as described, and the resulting TFA salt was then coupled to Boc-protected aminomethylbenzoic acid **4** (47 mg, 0,156 mmol, 1.2 equiv.) following general procedure A. Compound **8** was obtained in 44% yield after purification on column chromatography (DCM/MeOH, 94:6 v/v).

IUPAC-name: *tert*-butyl (*R*)-(4-((1-((1-((3-morpholinopropyl)amino)-1-oxo-3-phenylpropan-2-yl)carbamoyl)cyclopentyl)carbamoyl)benzyl)carbamate **8**; **Appearance**: white solid; **Molecular formula**: $C_{35}H_{49}N_5O_6$; **Molecular weight**: 635,81 g.mol^{-1}; **Yield**: 44% (37 mg); ^{1}**H-NMR** (300 MHz, $CDCl_3$) : 7.62 (d, J = 8.2 Hz, 2H), 7.34 (d, J = 8.2 Hz, 2H), 7.20-7.10 (m, 5H + NH), 6.62-6.55 (m, 2 x NH), 5.11-5.07 (m, NH), 4.67-4.60 (m, 1H), 4.36 (d, J = 6.1 Hz, 2H), 3.70-3.67 (m, 4 H), 3.30-3.23 (m, 2H), 3.21-3.08 (m, 2H), 2.47-2.32 (m, 7H), 2.09-1.64 (m, 9H), 1.47 (s, 9H); ^{13}**C-NMR** (75 MHz, $CDCl_3$): 173.0; 170.7; 167.8, 155.9; 143.8; 136.8; 132.4; 129.1; 128.5; 127.4; 127.3; 126.9; 79.9; 67.7; 66.7; 56.4; 54.1; 53.4; 44.2; 38.1; 37.7; 37.3; 36.4; 28.4; 25.4; 24.2; 24.1. **LC-MS** (ESI+) m/z calcd. for $C_{35}H_{50}N_5O_6$ $[M+H]^+$: 636.4, found : 636.5 , t_R = 22.4 min.(*system C*).

∇ **Synthesis of compound SBL-OPNK-2**

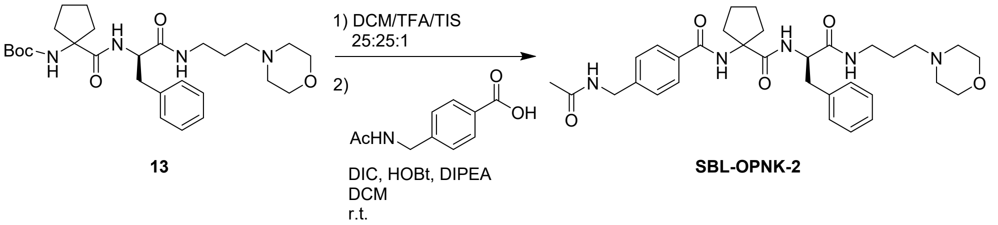

Following general procedure B, intermediate **13** (251 mg, 0.5 mmol, 1 equiv.) was first Boc-deprotected by treatment with DCM/TFA/TIS as described, and the resulting TFA salt was then coupled to *N*-acetyl-protected aminomethylbenzoic acid (106 mg, 0,55 mmol, 1.1 equiv.) following general procedure A. Crude compound was purified on semi-preparative HPLC, giving the product **SBL-OPNK-2** in 23% yield.

IUPAC-name: (*R*)-4-(acetamidomethyl)-N-(1-((1-((3-morpholinopropyl)amino)-1-oxo-3-phenylpropan-2-yl)carbamoyl)cyclopentyl)benzamide **SBL-OPNK-2**; **Appearance**: white solid; **Molecular formula**: $C_{32}H_{43}N_5O_5$; **Molecular weight**: 577.73 g.mol^{-1}; **Yield**: 23% (66 mg); ^{1}**H-NMR** (300 MHz, MeOD) : 7.82 (d, J = 8.4 Hz, 2H), 7.41 (d, J = 8.4 Hz, 2H), 7.25-7.15 (m, 5H), 4.56-4.49 (m, 1H), 4.44 (s, 2H), 4.09-4.01 (m, 2H), 3.78-3.68 (m, 2H), 3.47-3.41 (m, 3H), 3.29-2.98 (m, 7H), 2.37-2.28 (m, 1H), 2.03 (s, 3H), 2.01-1.65 (m, 9H) ; ^{13}**C-NMR** (75 MHz, MeOD): 176.8; 174.5; 173.3; 170.8; 144.7; 138.8; 133.9; 130.1; 129.5; 129.0; 128.4; 127.8; 68.6; 65.1; 56.5; 55.9; 53.2; 43.7; 38.0; 37.3; 37.1; 37.0; 32.5; 25.2; 24.7; 22.5; detailed characterization in table S1.

3.3. NK3 pharmacophore and hybrid synthesis procedures

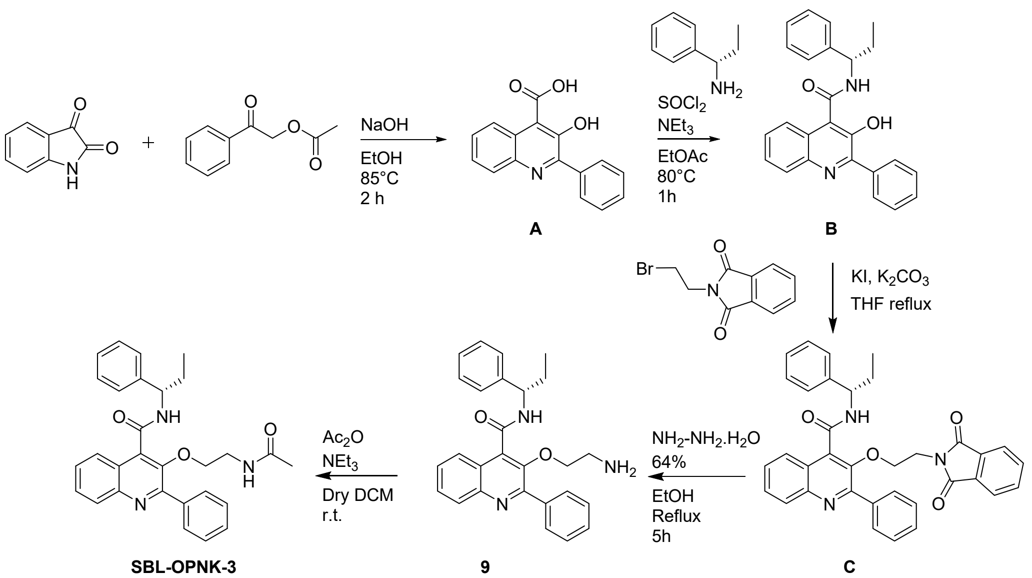

Scheme S 3 NK3 pharmacophore and hybrids synthetic pathway

Step 1: 3-Hydroxy-2-(2-methoxyphenyl)quinoline-4-carboxylic acid intermediate **A** was prepared according to reported procedure [4,5]: a solution of acetoxy-2'-methoxyacetophenone (3.8 g, 21 mmol, 1.5 equiv.) in EtOH (20 mL), heated and stirred for 5 minutes at 85°C, was added to a solution of isatin (2 g, 13.5 mmol, 1 equiv.) and 10 N NaOH (12 mL) in EtOH (9.2 mL) at 85°C. After the mixture was stirred at 85°C for 2 h, H_2O (9.0 mL) was added to the reaction mixture at 0°C. The mixture was acidified to pH 1 with 1 N HCl. The precipitate was collected by filtration and washed with H_2O and EtOH to give the title compound as a bright yellow solid (quantitative yield). Analytical data are consistent with literature [5].

Step 2: Intermediate **B** was prepared according following an adapted version of a reported procedure [5]: To a solution of **A** (1.8 g, 6.8 mmol, 1 equiv.) in EtOAc (43 mL), was added dropwise at 0°C triethylamine (TEA) (2.8 mL, 3 mmol, 3 equiv.) followed by thionyl chloride (0.5 mL, 6.8 mmol, 1 equiv.). The mixture was let to warm up to room temperature for 1h. α-(*S*)-ethylbenzylamine was then added to the flask, and the mixture was heated at 80°C for 1.5 h, before being cooled to room temperature. The solution was then evaporated to dryness, and the crude product was solubilized in dichloromethane. The resulting crude mixture was then successively washed with water, 5% aqueous solution of citric acid, 5% aqueous solution of $NaHCO_3$, and finally brine. After drying on $MgSO_4$, the resulting layer was evaporated under reduced pressure to give the titled compound **B** as a bright yellow solid in 67% yield (1.7 g). Analytical data were consistent with literature [5].

Step 3: Following a reported procedure [5], compound **B** (5.0 mmol) was dissolved in dry THF (20 mL). Separately, a mix of *N*-(2-bromoethyl)phthalimide (25.0 mmol, 5 equiv.), K_2CO_3 (15.0 mmol), and KI (1.5 mmol) were dissolved in dry THF (15 mL), and subsequently added to compound B solution. The reaction mixture was stirred at room temperature for 2.5 h and then refluxed for 10 h; K_2CO_3 was filtered off, and the mixture was evaporated to dryness, dissolved in CH_2Cl_2, and washed with H_2O. The organic layer was dried over Na_2SO_4 and evaporated to dryness. The residue was flash chromatographed on silica gel, with EtOAc/cyclohexane (1:3) eluant to give the title compound **C** in 60% yield. Analytical data are consistent with literature [5].

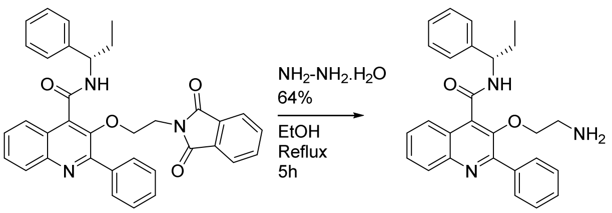

C **9**

Step 4: Phthalimidomethyl-phenylquinoline **C** (278 mg, 0.5 mmol, 1 equiv.) was suspended in EtOH 96% (25 mL). Hydrazine hydrate 64% (0.123 mL, 2.5 mmol, 5 equiv.) was added, and the reaction mixture was refluxed until complete conversion (~5h). The reacting mixture was evaporated to dryness, then the resulting crude was solubilized in 20 mL H_2O, cooled and acidified with 37% HCl (10 mL). The mixture was refluxed for 45 min and cooled. The precipitated phthalhydrazide was filtered off and the aqueous filtrate was made alkaline with 40% NaOH and eventually extracted with CH_2Cl_2. The organic layer was washed with brine, separated, dried over Mg_2SO_4, and evaporated to dryness. The residue was flash chromatographed on 230-400 mesh silica gel, eluting with CH_2Cl_2/MeOH and 1% NEt_3 (96:4) to give the title compound **9** in quantitative yield as a yellow solid. Analytical data are consistent with literature [5].

IUPAC-name: (*S*)-3-(2-aminoethoxy)-2-phenyl-*N*-(1-phenylpropyl)quinoline-4-carboxamide **9**; **Appearance**: yellow solid; **Molecular formula**: $C_{27}H_{27}N_3O_2$; **Molecular weight**: 425.53 g.mol^{-1}; **Yield**: quantitative (0.21 g); ^{1}**H-NMR** (300 MHz, $CDCl_3$) : 8.10 (d, J = 8 Hz, 1H); 7.91-7.88 (m, 2H), 7.81 (dd, J_1 = 8.3 Hz, J_2 = 0.8 Hz, 2H); 7.67-7.61 (m, 1H), 7.53-7.29 (m, 10H), 7.00 (d, J = 8.0 Hz; 1 H), 5.28-5.20 (m, 1H), 3.60-3.57 (m, 2H); 2.64 (m, 2H), 2.06-1.90 (m, 2H), 1.50 (m, 3H), 1.04 (t, J = 7.4 Hz, 3H); ^{13}**C-NMR** (75 MHz, $CDCl_3$): 165.6, 161.1, 153.2, 146.1, 143.4, 141.2, 135.2, 135.0, 130.4, 130.2, 129.0, 128.9, 128.6, 128.1, 128.0, 126.9, 124.4, 123.9, 70.4, 56.7, 40.0, 28.8, 10.8.

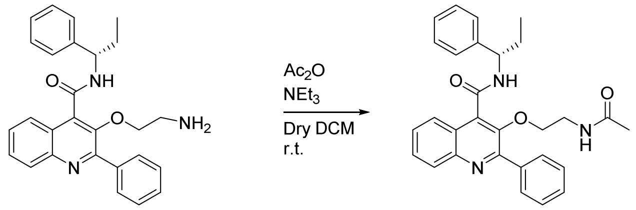

9 **SBL-OPNK-3**

Step 5: Compound **9** (106 mg, 0.25mmol, 1 equiv.) was solubilized in dry THF, with acetic anhydride (0.421 mL, 2.5 mmol, 10 equiv.) and NEt_3 (0.038 mL, 0.25 mmol, 1 equiv.). Mixture was stirred for 48h at room temperature. After reaction completion, solvent was evaporated and crude product was chromatographed with CH_2Cl_2/MeOH (97:3) to give the title compound **SBL-OPNK-3** in quantitative yield.

IUPAC-name: (*S*)-3-(2-acetamidoethoxy)-2-phenyl-N-(1-phenylpropyl)quinoline-4-carboxamide **SBL-OPNK-3**; **Appearance**: yellow solid; **Molecular formula**: $C_{29}H_{29}N_3O_3$; **Molecular weight**: 467.57 g.mol^{-1}; **Yield**: quantitative (116 mg); ^{1}**H-NMR** (300 MHz, $CDCl_3$) : 8.12 (d, J = 8.3 Hz, 1H), 7.86-7.83 (m, 2H), 7.77-7.73 (m, 2H), 7.70-7.64 (m, 1H), 7.58-7.55 (m, 1H), 7.52-7.48 (m, 3H), 7.45-7.37 (m, 4H), 7.35-7.30 (m, 1H), 7.07 (d, J = 8.2 Hz, 1H), 6.52-6.48 (m, 1H), 5.25-5.18 (m, 1H), 3.67-3.51 (m, 2H), 3.18-2.99 (m, 2H), 2.04-1.94 (m, 2H), 1.69 (s, 3H), 1.06 (t, J = 7.4 Hz, 3H); ^{13}**C-NMR** (75 MHz, $CDCl_3$): 171.2; 165.4; 153.9; 146.6; 143.3; 141.5; 135.9; 135.5; 130.0; 129.7; 129.1; 128.8; 128.8; 128.3; 128.0; 127.8; 126.6; 124.9; 124.1; 73.9; 56.2; 39.5; 29.3; 22.3; 11.0. See further characterization in table S1

4. NMR spectra and HPLC chromatographs

∇ Compound 12

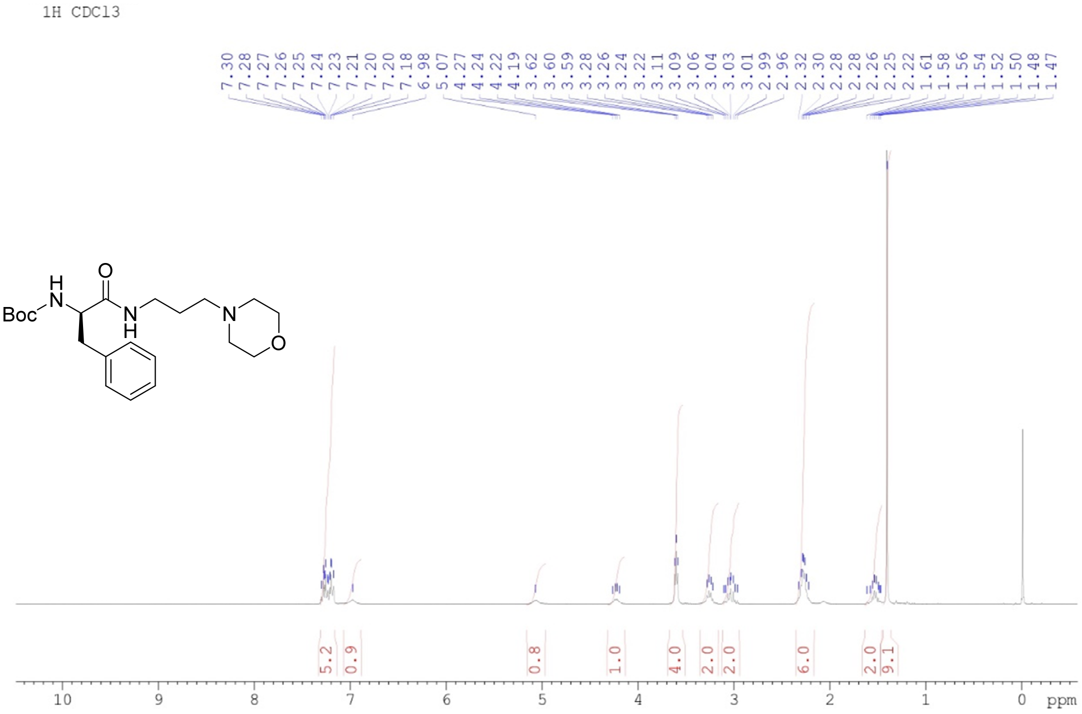

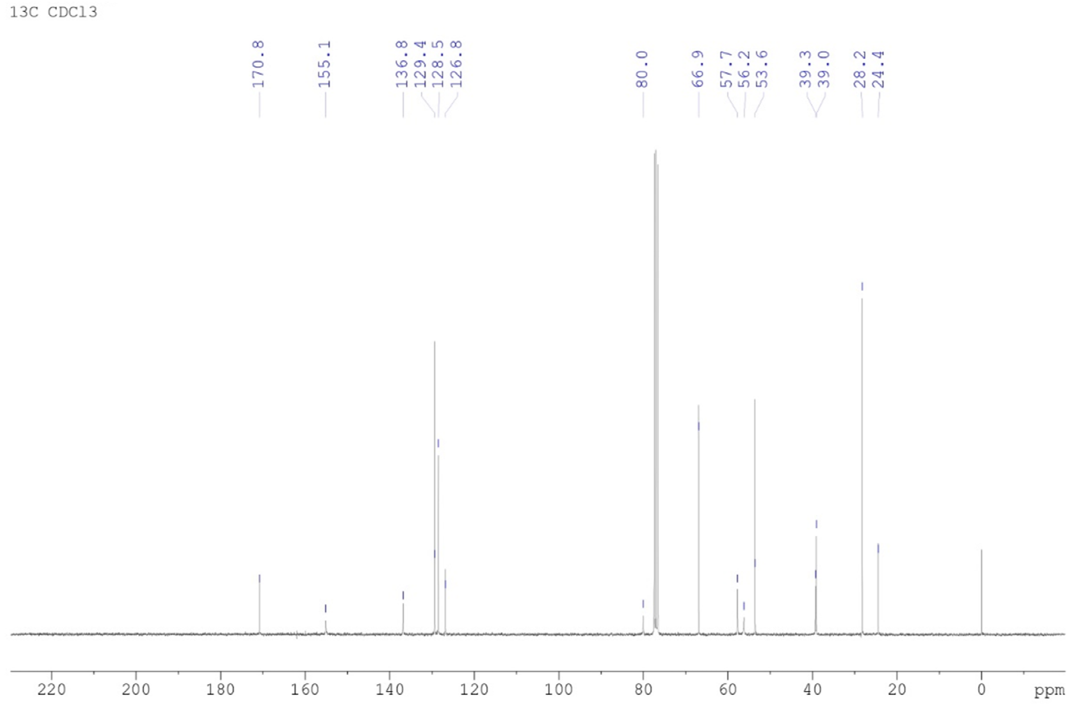

LC Chromatograph 1 compound 12 *(System B)*

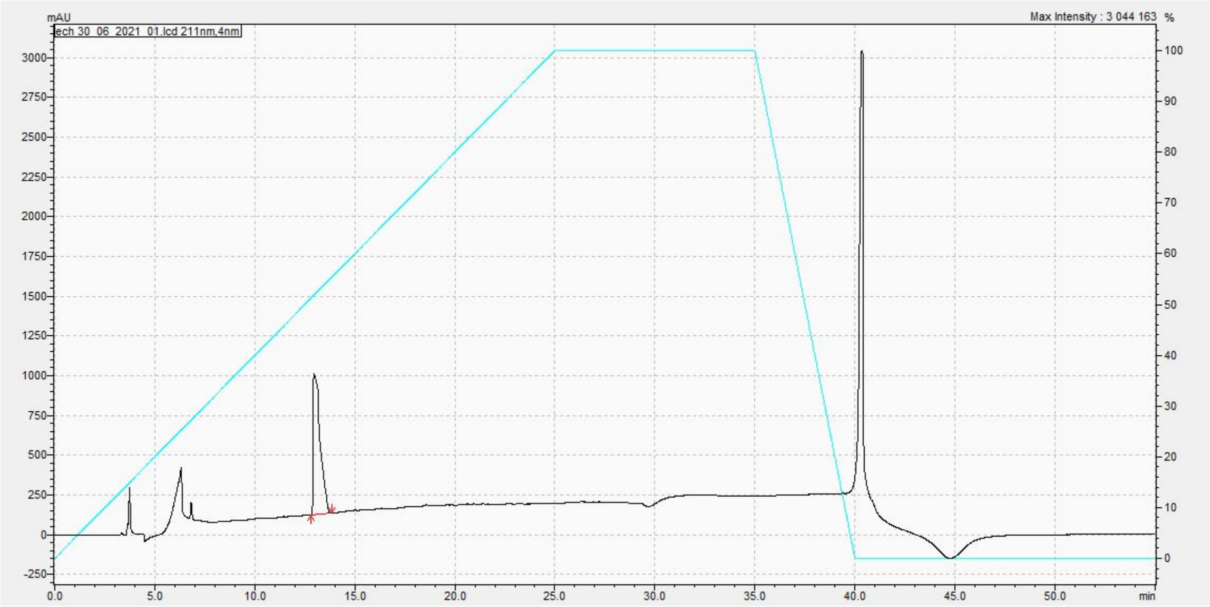

∇ Compound 13

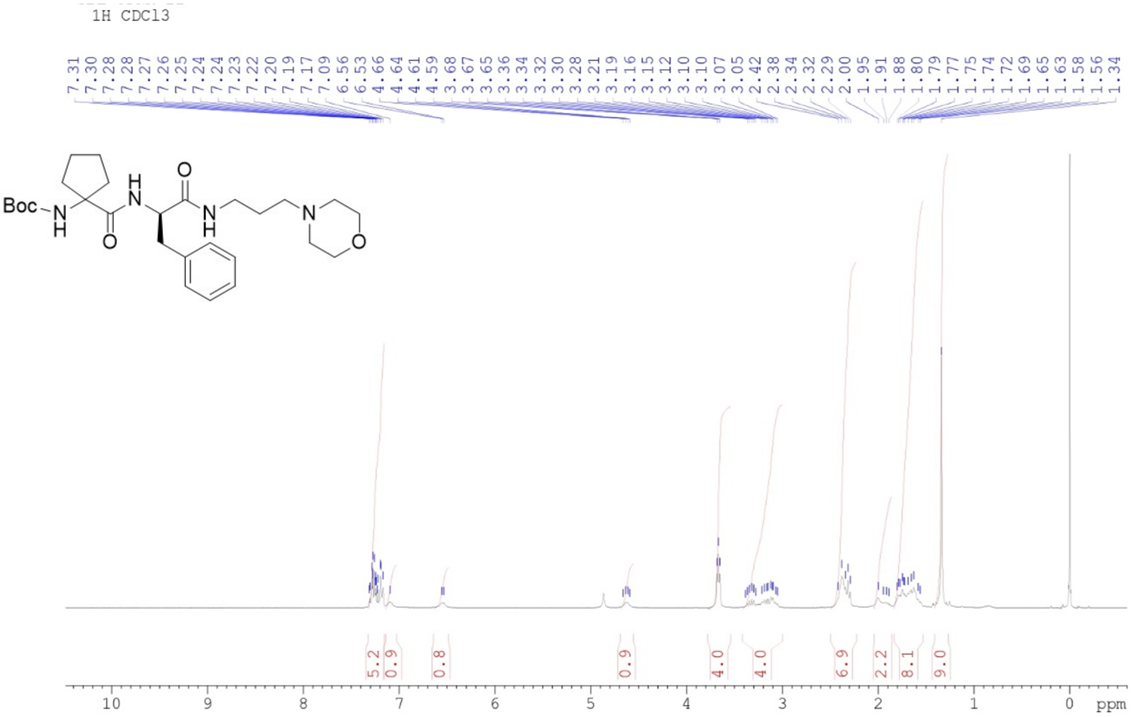

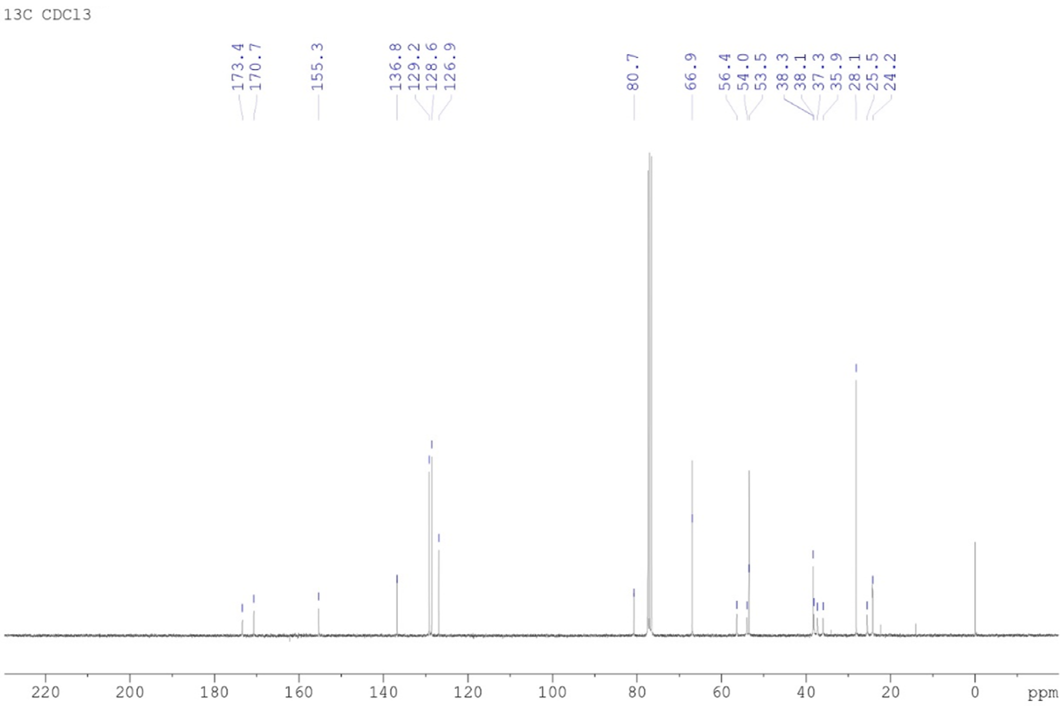

LC Chromatograph 2 compound 13 *(System B)*

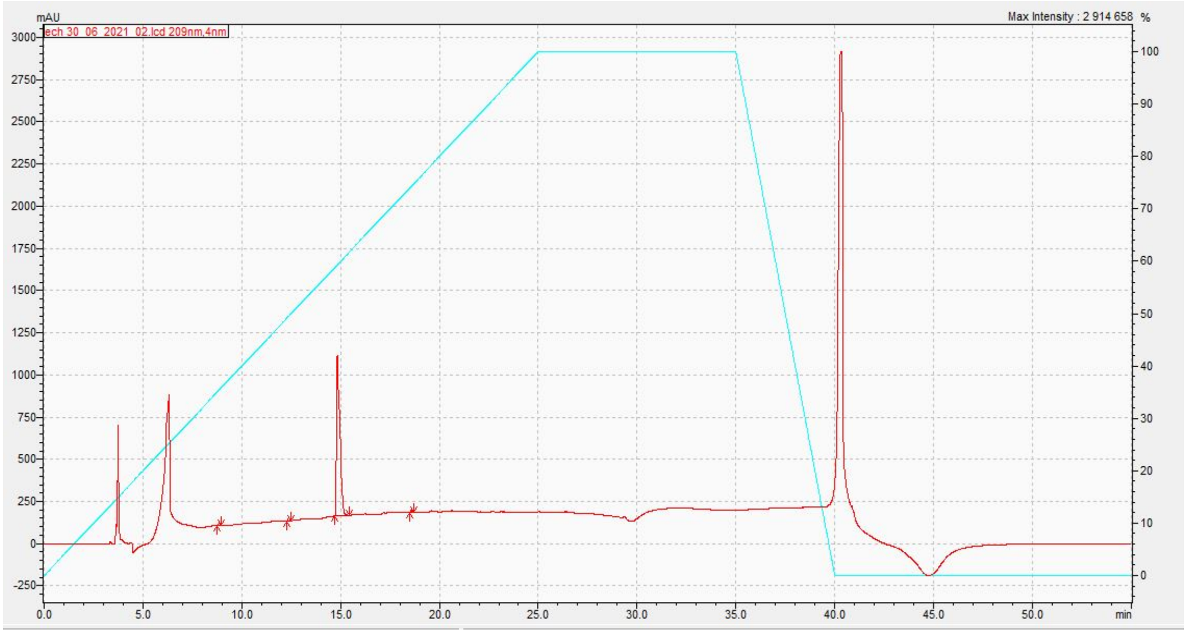

∇ Compound 8

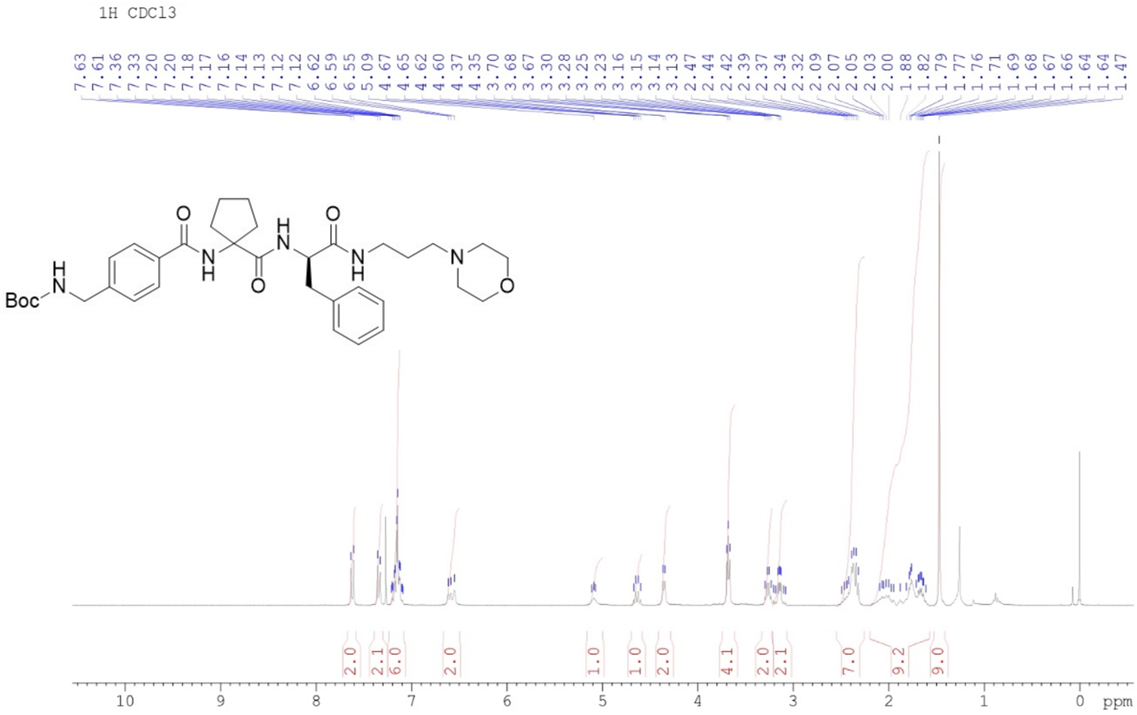
1H CDCl3
7.63 7.61 7.36 7.33 7.20 7.20 7.18 7.17 7.16 7.14 7.13 7.12 6.62 6.59 6.55 5.09 4.67 4.65 4.62 4.60 4.37 4.35 3.70 3.68 3.67 3.30 3.28 3.25 3.23 3.16 3.15 3.14 3.13 2.47 2.44 2.42 2.39 2.37 2.34 2.32 2.09 2.07 2.05 2.03 2.00 1.88 1.82 1.79 1.77 1.76 1.71 1.69 1.68 1.67 1.66 1.64 1.64 1.47
Boc
N
H
O
N
H
O
H
N
O
N
H
N
O
2.0 2.1 6.0 2.0 1.0 1.0 2.0 4.1 2.0 2.1 7.0 9.2 9.0
10 9 8 7 6 5 4 3 2 1 0 ppm

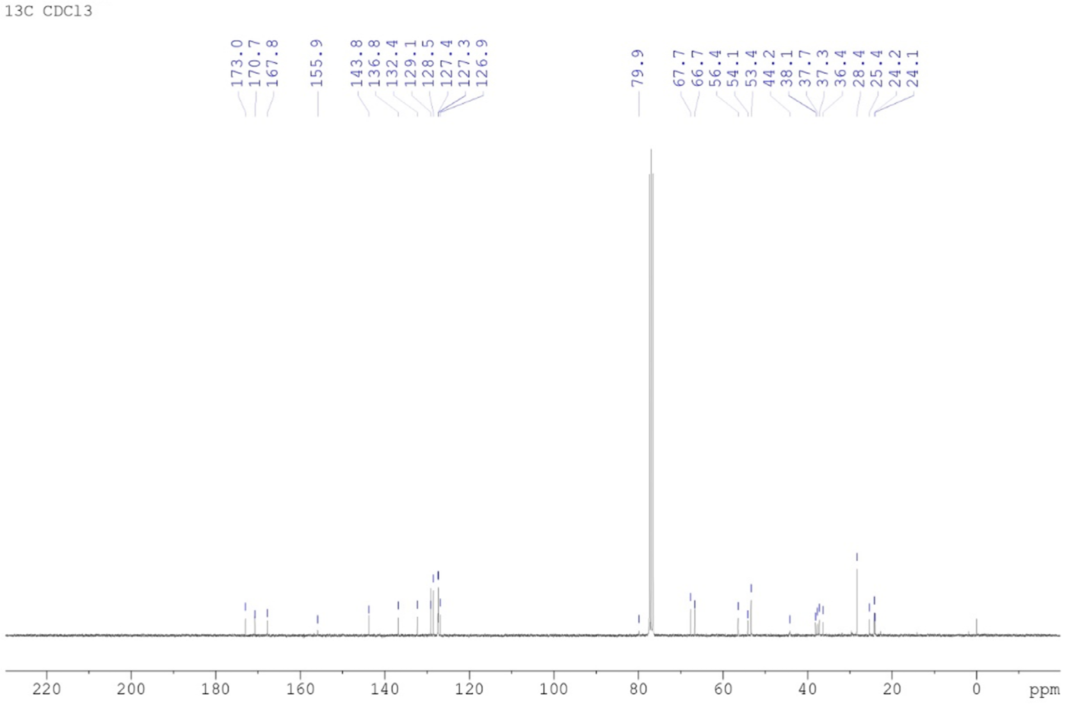
13C CDCl3
173.0 170.7 167.8 155.9 143.8 136.8 132.4 129.1 128.5 127.4 127.3 126.9 79.9 67.7 66.7 56.4 54.1 53.4 44.2 38.1 37.7 37.3 36.4 28.4 25.4 24.2 24.1
220 200 180 160 140 120 100 80 60 40 20 0 ppm

LC Chromatograph 3 Compound 8 *(System B)*

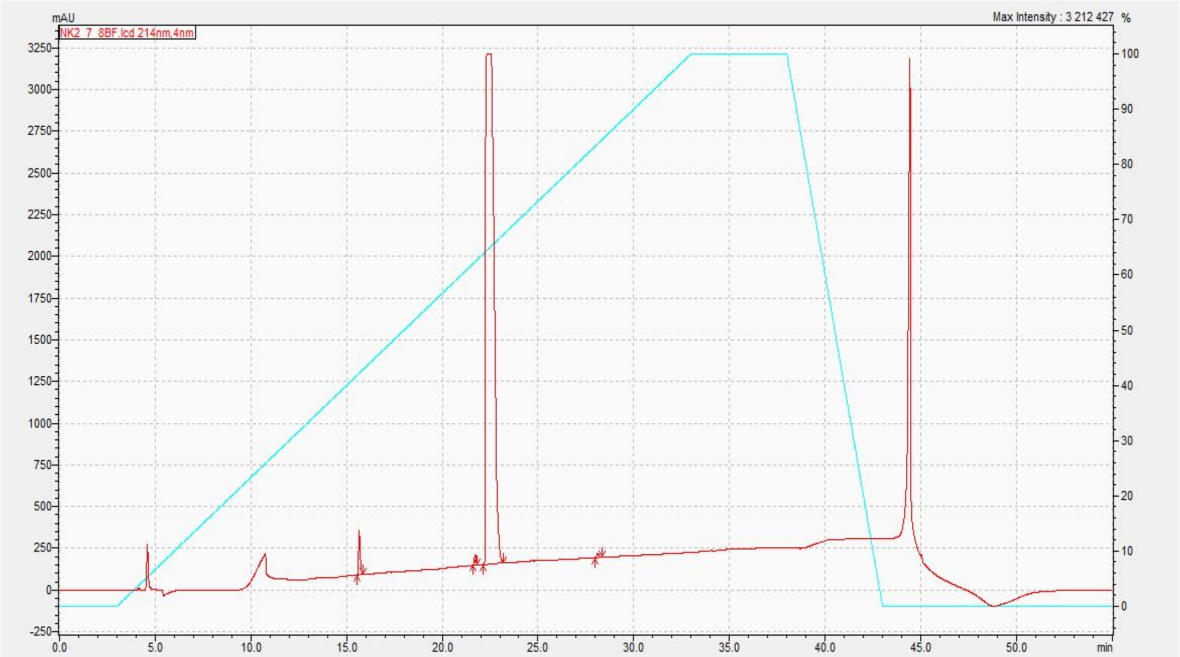

∇ SBL-OPNK-2

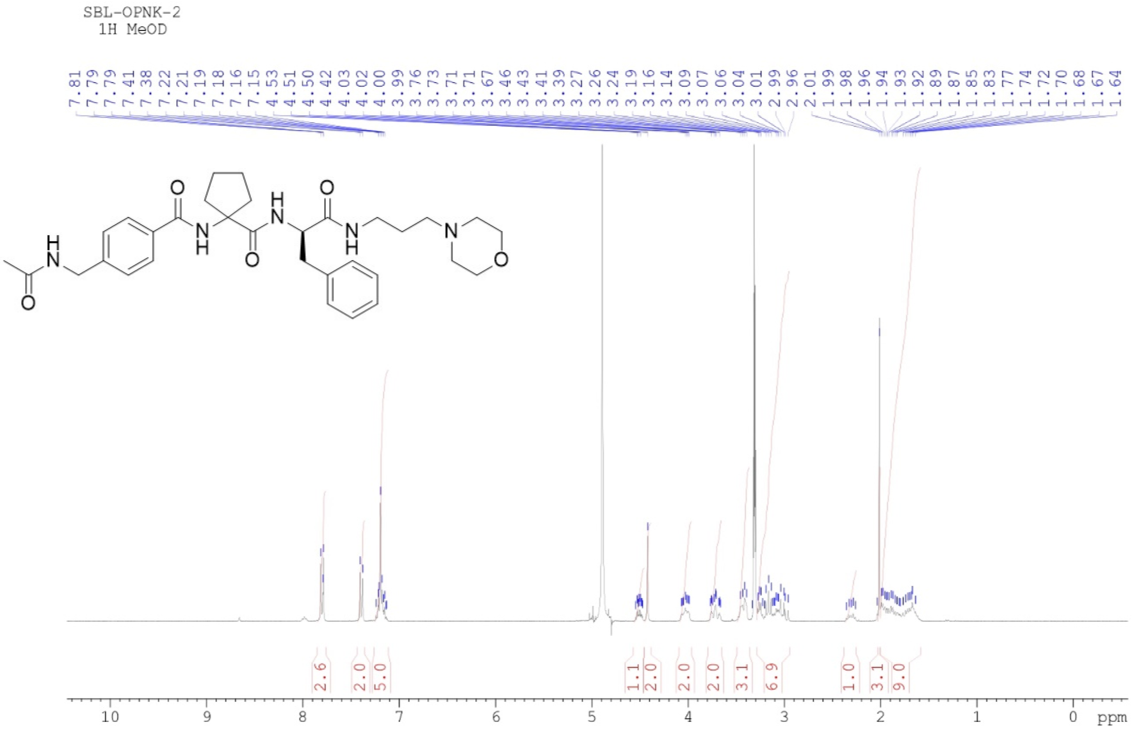

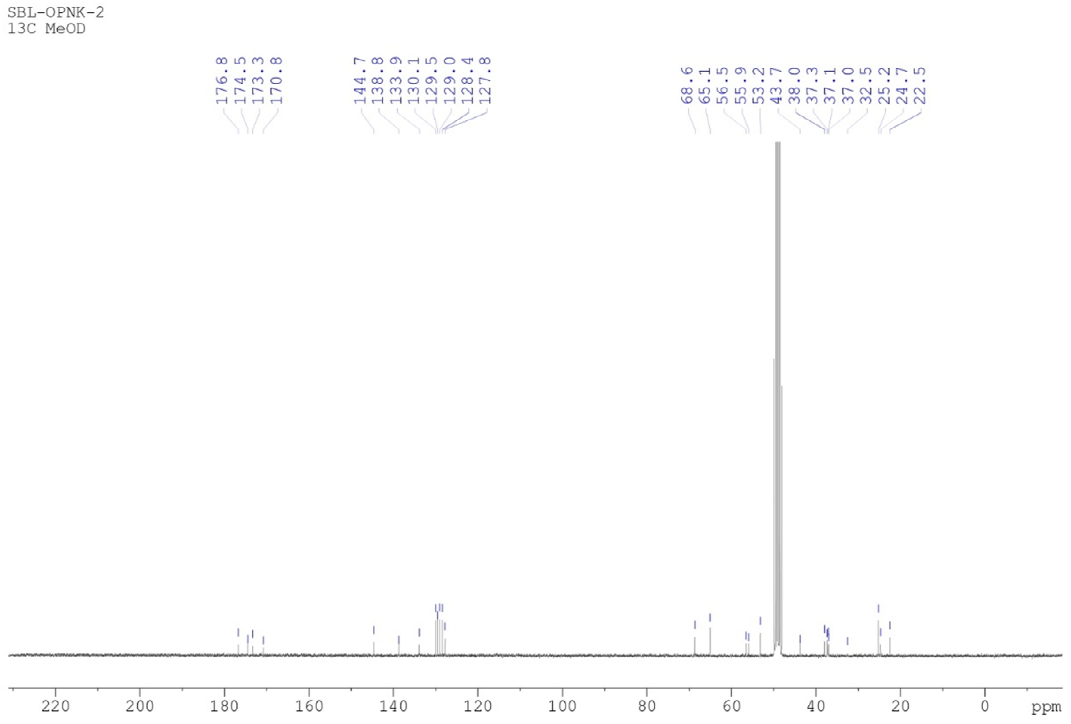

LC Chromatograph 4 SBL-OPNK-2 *(System B)*

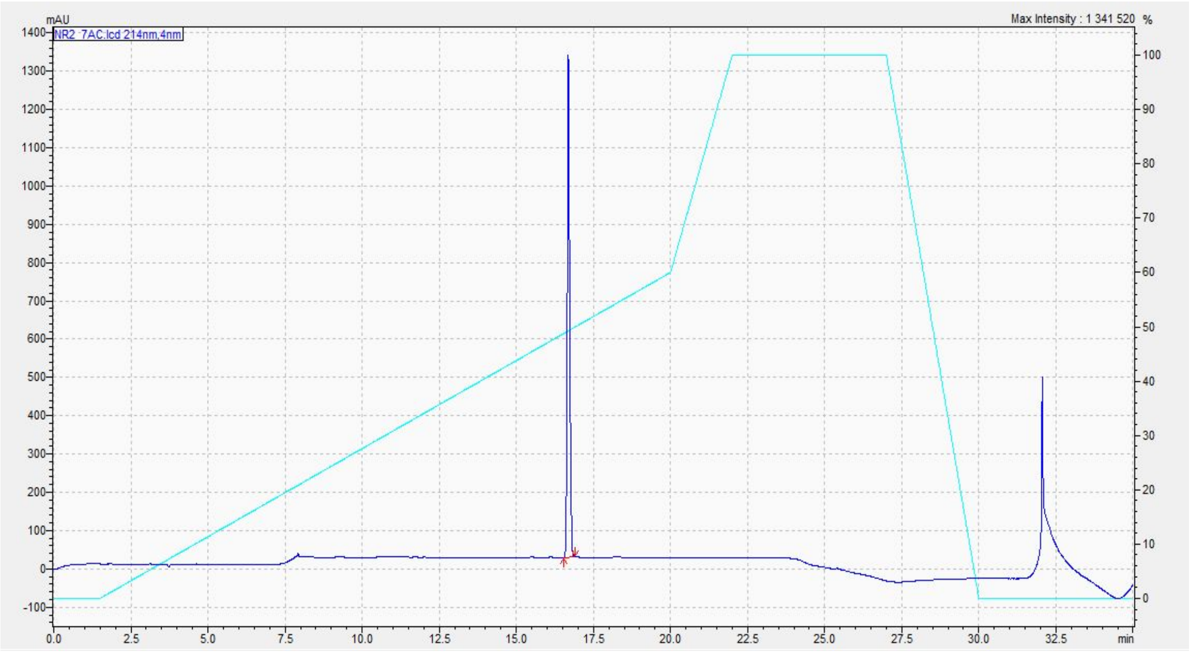

∇ Compound 9

1H CDCl3

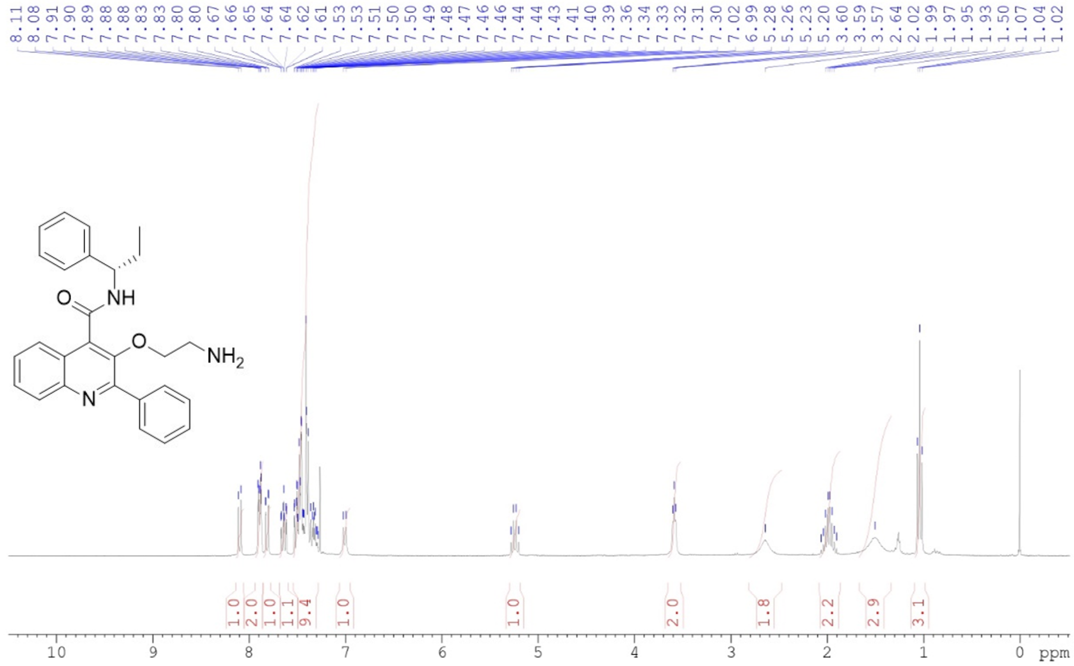

13C CDCl3

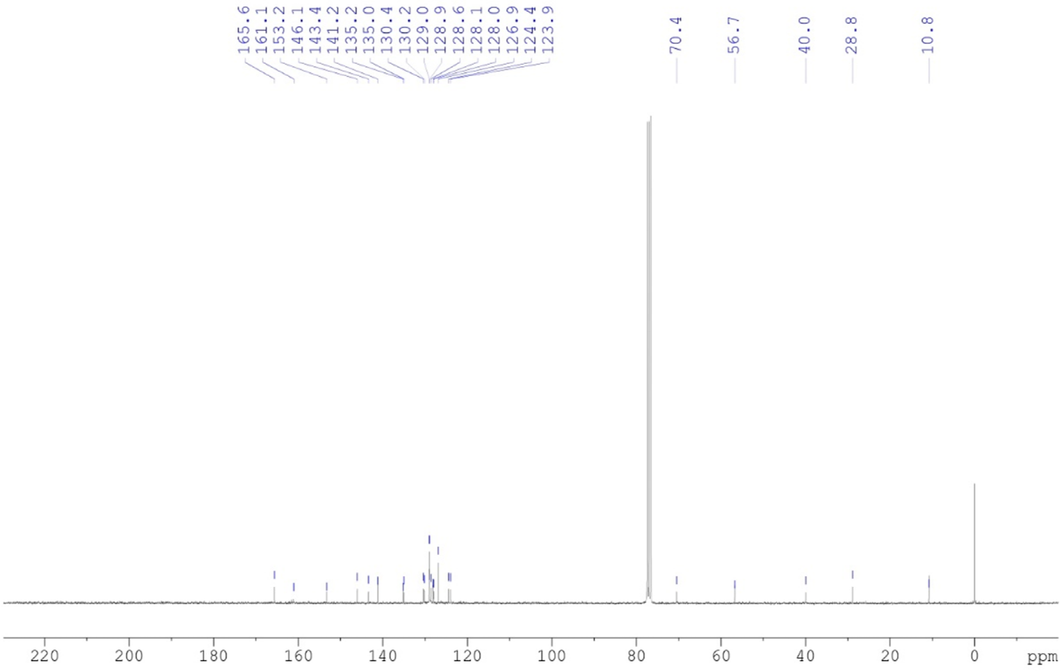

LC Chromatograph 5 Compound 9 *(System B)*

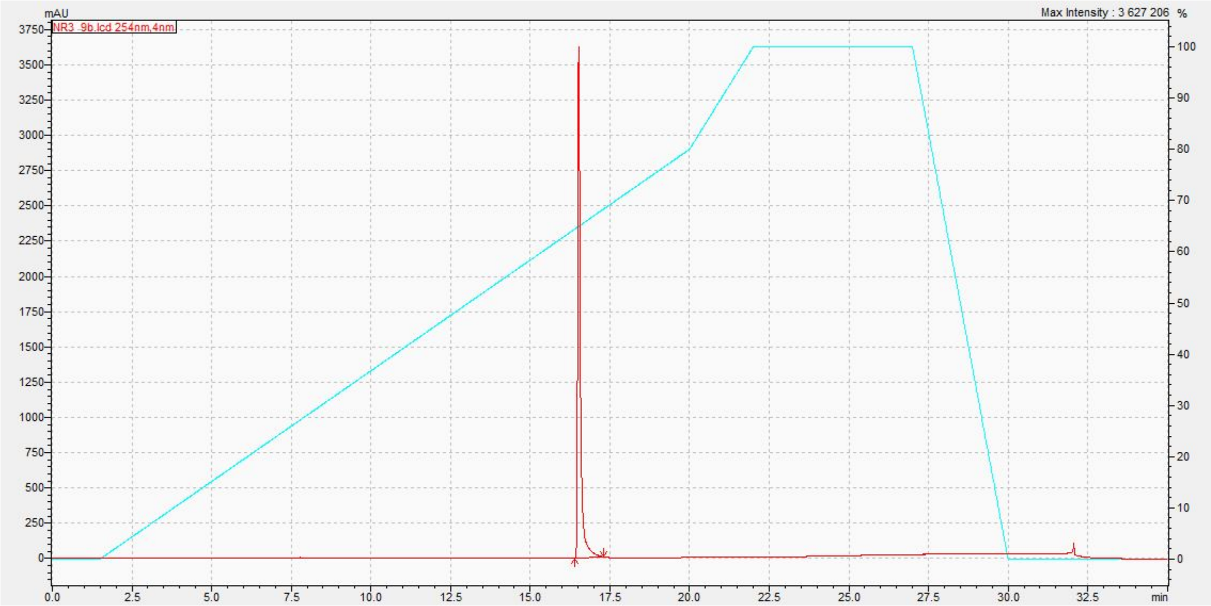

∇ SBL-OPNK-3

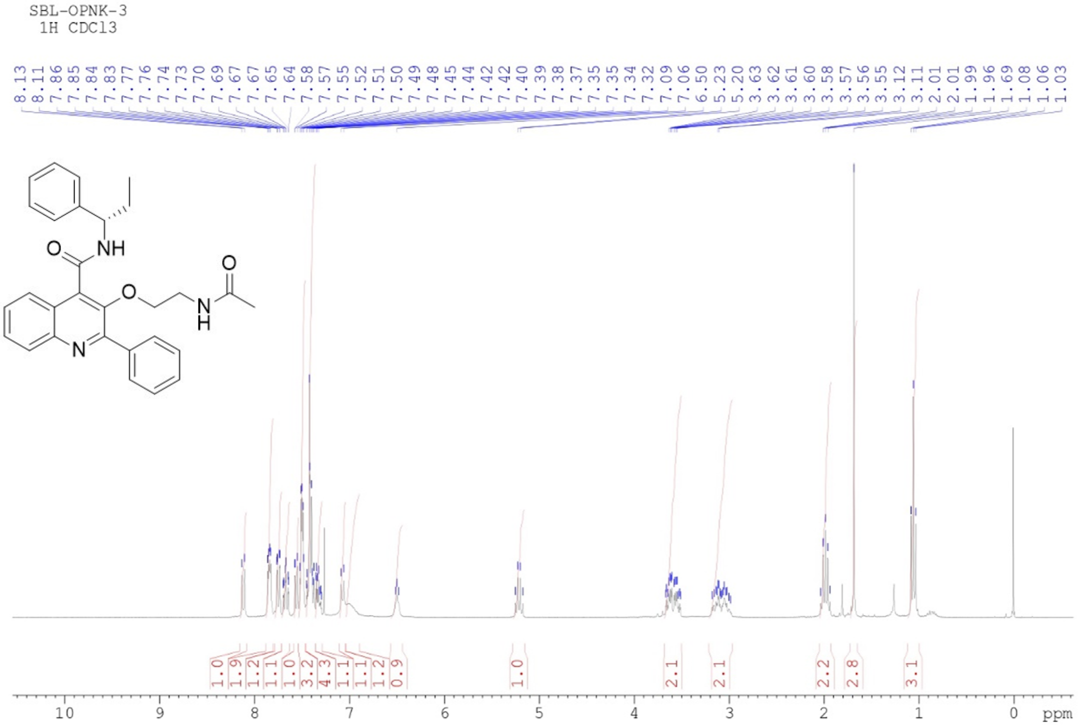

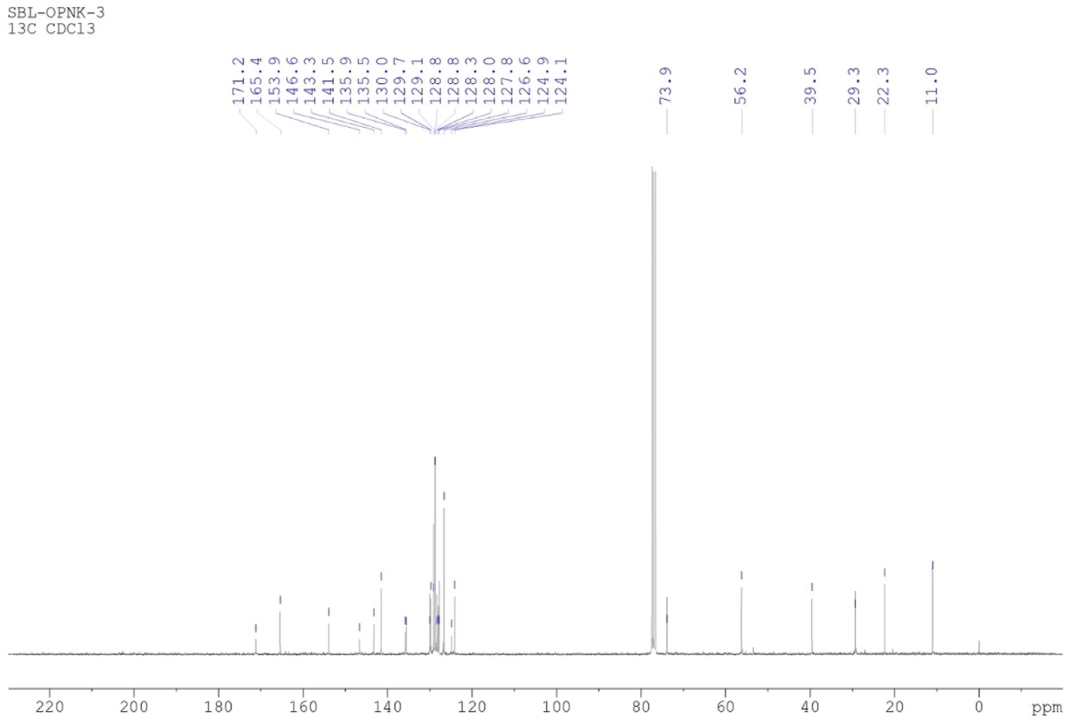

LC Chromatograph 6 SBL-OPNK-3 *(System B)*

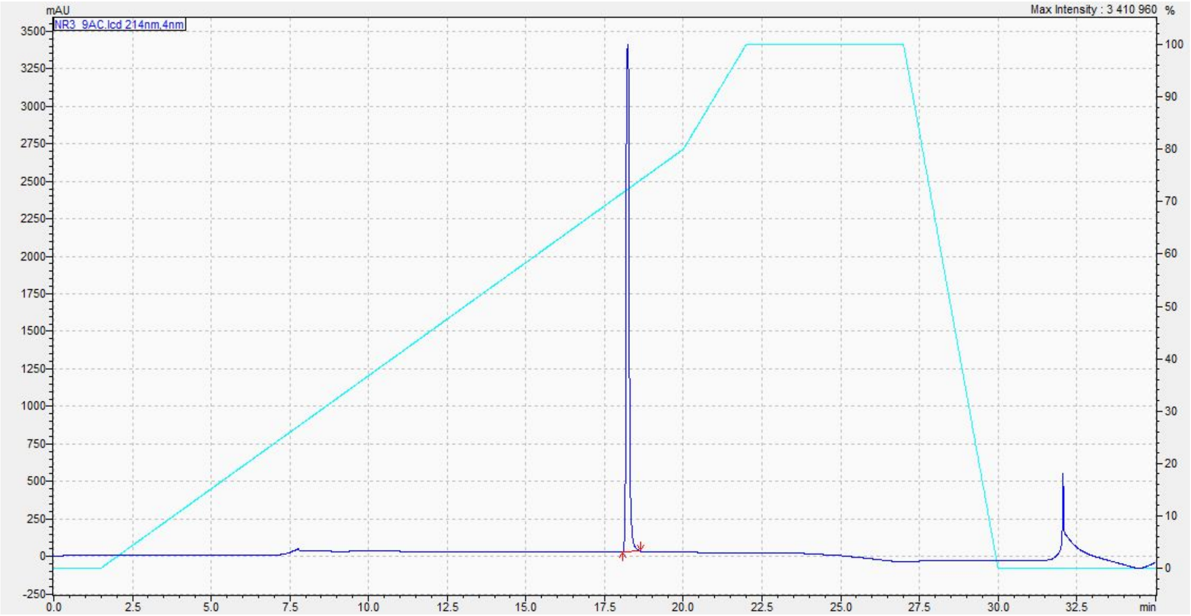

5. Correlation between radioligand binding and calcium mobilization assays for MOR and DOR

The coefficient of determination equals 0.97 and 0.96 for MOR and DOR, respectively, indicating a good correlation between radioligand binding assays and calcium mobilization test (Figure S1).

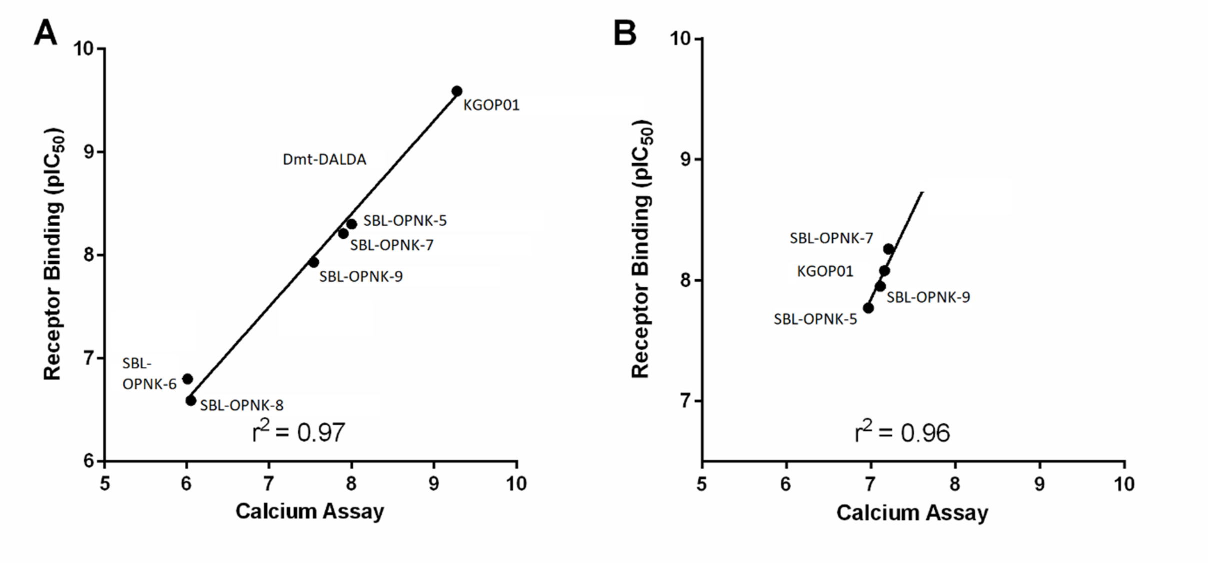

Figure S1. Correlation between Radioligand Binding and Calcium Mobilization assays for MOR (Panel A) and DOR (Panel B).

References:

1. Song, X.; Xu, C.R.; He, H.T.; Siahaan, T.J. Synthesis of a Novel Cyclic Prodrug of RGD Peptidomimetic to Improve Its Cell Membrane Permeation. *Bioorganic Chem.* **2002**, *30*, 285–301, doi:10.1016/S0045-2068(02)00013-5.
2. Garcia, J.; Mata, E.G.; Tice, C.M.; Hormann, R.E.; Nicolas, E.; Albericio, F.; Michelotti, E.L. Evaluation of Solution and Solid-Phase Approaches to the Synthesis of Libraries of α,α-Disubstituted-α-Acylaminoketones. *J. Comb. Chem.* **2005**, *7*, 843–863, doi:10.1021/cc0500396.
3. Fedi, V.; Altamura, M.; Catalioto, R.-M.; Giannotti, D.; Giolitti, A.; Giuliani, S.; Guidi, A.; Harmat, N.J.S.; Lecci, A.; Meini, S.; et al. Discovery of a New Series of Potent and Selective Linear Tachykinin NK2 Receptor Antagonists. *J. Med. Chem.* **2007**, *50*, 4793–4807, doi:10.1021/jm070289w.
4. Yamamoto, K.; Okazaki, S.; Ohno, H.; Matsuda, F.; Ohkura, S.; Maeda, K.; Fujii, N.; Oishi, S. Development of Novel NK3 Receptor Antagonists with Reduced Environmental Impact. *Bioorg. Med. Chem.* **2016**, *24*, 3494–3500, doi:10.1016/j.bmc.2016.05.054.
5. Giardina, G.A.M.; Raveglia, L.F.; Grugni, M.; Sarau, H.M.; Farina, C.; Medhurst, A.D.; Graziani, D.; Schmidt, D.B.; Rigolio, R.; Luttmann, M.; et al. Discovery of a Novel Class of Selective Non-Peptide Antagonists for the Human Neurokinin-3 Receptor. 2. Identification of (S)-N-(1-Phenylpropyl)-3-Hydroxy-2- Phenylquinoline-4-Carboxamide (SB 223412). *J. Med. Chem.* **1999**, *42*, 1053–1065, doi:10.1021/jm980633c.
